# Effectiveness of Shuxuening injection in coronary heart disease: a systematic review and meta-analysis

**DOI:** 10.3389/fphar.2023.1265603

**Published:** 2023-09-18

**Authors:** Menglong Shi, Tianye Sun, Zhaochen Ji, Yucong Ma, Min Zhao, Fengwen Yang, Junhua Zhang

**Affiliations:** ^1^ State Key Laboratory of Component-Based Chinese Medicine, College of Traditional Chinese Medicine, Tianjin University of Traditional Chinese Medicine, Tianjin, China; ^2^ Evidence-Based Medicine Center, College of Traditional Chinese Medicine, Tianjin University of Traditional Chinese Medicine, Tianjin, China; ^3^ Dongfang Hospital, Beijing University of Chinese Medicine, Beijing, China; ^4^ The First Affiliated Hospital of Henan University of CM, Zhengzhou, China

**Keywords:** Shuxuening injection, coronary heart disease, meta-analysis, systematic review, randomized controlled trial

## Abstract

**Background:** Coronary heart disease (CHD) poses a serious threat to public health, and the current medical management still faces significant challenges. Reliable evidence on the efficacy of Shuxuening injection (SXNI) in CHD is still lacking, even though it is widely used in China.

**Purpose:** To evaluate the efficacy of SXNI combination therapy in treating CHD.

**Methods:** A systematic search of eight databases was conducted to identify relevant randomized controlled trials (RCTs) from the inception of each database until June 2023. ROB 2.0, RevMan 5.4, and Stata 15.1 were used for quality evaluation and data analysis. The Grading of Recommendation, Assessment, Development, and Evaluation (GRADE) approach was used to evaluate the quality of evidence.

**Results:** A total of 3,779 participants from 39 studies were included. The results showed SXNI combination therapy increased the clinical efficacy and decreased the frequency and duration of angina. Furthermore, SXNI combination therapy improved cardiac function of patients by decreasing LVEDD, and increased CI, CO, and LVEF. It also improved blood lipid profiles by increasing HDL, decreasing TC, TG, and LDL. The thrombosis factors of patients were also improved by decreasing FIB, PV, HCT, and HS. Moreover, SXNI combination therapy was superior to the conventional treatment in improving CRP levels, increasing ECG efficacy and BNP. However, due to the limited safety information, reliable safety conclusions could not be drawn. Furthermore, the levels of evidence ranged from very low to moderate due to publication bias and heterogeneity.

**Conclusion:** SXNI can effectively improve angina symptoms, clinical efficacy, cardiac function, blood lipid indicators, and thrombosis factors of patients with CHD. However, more multi-center and large-sample studies are needed to confirm the conclusions due to the limitations of this study.

**Registration** https://www.crd.york.ac.uk/prospero/display_record.php?RecordID=399606; Identifier: CRD42023433292.

## Graphical Abstract

**Figure:**
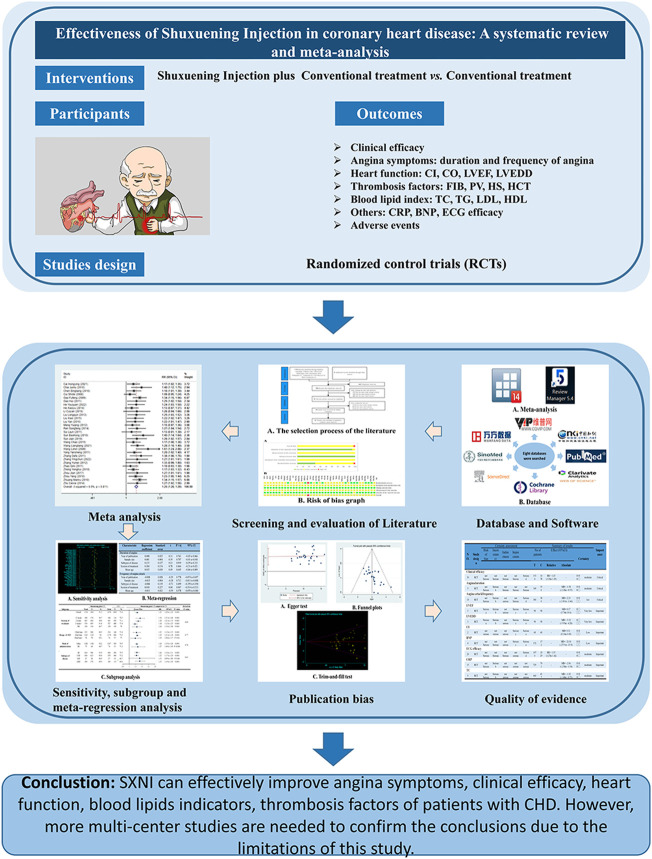

## 1 Introduction

Coronary heart disease (CHD) is a major cardiovascular disease that poses a serious threat to public health. In 2019, it resulted in approximately 9 million deaths worldwide (World Health Organization, 2020). CHD is characterized by stenosis or occlusion of blood vessels due to coronary atherosclerosis and leads to myocardial ischemia, hypoxia, and necrosis (Boudoulas et al., 2016). There are about 11 million patients with CHD in China, of which the mortality rate of CHD in urban residents is 126.91 per 100,000, and that of rural residents is 135.88 per 100,000 (Su et al., 2023). CHD can also have detrimental effects on the nervous system, endocrine system, and mental health (Shmakova et al., 2022). Epidemiological studies have found that nearly 31% of patients with CHD have anxiety and depression (Palacios et al., 2018). In addition, as a chronic disease, CHD places a significant economic burden on individuals and society (GBD, 2016 DALYs and HALE Collaborators, 2017). Data shows that the medical management of CHD is one of the most expensive components of the healthcare system in China, with total CHD-related hospitalization costs of approximately 11.2 billion yuan in 2018 (Ma et al., 2020).

The current management mode of patients with CHD is medical management and hierarchical prevention (Shmakova et al., 2022). The primary object of drug therapy is to alleviate symptoms of angina pectoris and reduce the occurrence of cardiovascular events. Commonly prescribed medications for CHD include antiplatelets, statins, beta-blockers, and nitrates (Guo and Zhong, 2018). Despite advancements in drug management, the pharmacological management of CHD remains a challenge due to limited effectiveness and issues with patient compliance (Joshi and de Lemos, 2021). Research indicates that the hospitalization rate for acute myocardial infarction in China has been increasing annually. However, there has not been a significant reduction in in-hospital mortality (Zhu et al., 2023). Therefore, it is crucial to explore effective interventions to enhance the treatment of CHD.

Based on available clinical evidence, traditional Chinese medicine is increasingly recognized for its significant role in the treatment of CHD and its complications (Liu et al., 2022; Liu et al., 2023; Shen et al., 2023; Tao et al., 2023). Shuxuening injection (SXNI), a sterile preparation from folium Ginkgo [Yinxingye, Ginkgo biloba L.], is an botanical drug injection approved by the Chinese State Food and Drug Administration. The main active ingredients are total flavonoid glycosides and ginkgolide. The botanical drugs included and traditional effects of Shuxuening injection was provided in Supplementary Table S2. Pharmaceutical manufacturer obtained Ginkgo biloba leaf, and an appropriate amount of water was added for injection to dissolve it, followed by activated carbon. After homogenizing and filtering, water was injected into the specified volume. Then, 1% shydrochloric acid was added to adjust the pH value to 3.8–4.0, followed by filtering, and filling, to obtain SXNI. The chemical content of one injection of SXNI (5 mL/unit) containing 4.2 mg total Ginkgo flavonol glycosides and 0.7 mg Ginkgolides, is equal to 17.5 mg Ginkgo biloba leaf extract according to manufacturer’s instruction. Studies have proven SXNI to be effective in improving coronary blood supply, reducing myocardial infarct size, and improving myocardial pathological damage (Li et al., 2019; Yu et al., 2019). Additionally, pharmacological studies have revealed that SXNI can effectively protect cardiomyocytes, reduce myocardial damage, reduce platelet adhesion rate and enhance cellular immune activity (Wang et al., 2019). Furthermore, SXNI has shown an ability to reduce the expression of nuclear factor kappa B p65 (NF-κBp65), inducible nitric oxide synthase (iNOS), and monocyte chemoattractant protein-1 (MCP-1). This effect significantly improves vascular endothelial function and delays the progression of atherosclerosis and thrombosis (Xu et al., 2009). Clinical studies have demonstrated that SXNI improves cardiac function, quality of life, daily living ability, and myocardial infarct size in patients with CHD (He et al., 2022).

With the wide application of SXNI and the growing number of clinical studies, the number of systematic reviews (SRs) assessing its effectiveness and safety has also increased (Zhou et al., 2015; Wu et al., 2017; Fan et al., 2022). However, an umbrella review of these SRs has identified several shortcomings. First, the potential heterogeneity and publication bias were not analyzed and explained using subgroup, sensitivity, meta-regression, or trim-and-fill analyses. Second, there was an absence of quality evaluation of the evidence. Moreover, due to the limited number of outcome indicators, the efficacy and safety of SXNI were not comprehensively evaluated in the reviewed SRs. These limitations reduce the credibility of the evidence and its applicability in clinical practice.

This study will strictly follow the Preferred Reporting Program for Systematic Review and Meta-Analysis (PRISMA) (Page et al., 2021), avoid limitations of previous SRs, and update the evidence regarding the efficacy and safety of SXNI injection in treating CHD.

## 2 Methods

This review has been registered on The International Prospective Register of Systematic Reviews (PROSPERO) (CRD42023433292) (Page et al., 2018).

### 2.1 Search strategy

Two researchers (SML and STY) independently searched all relevant studies up to June 2023 from four English databases (PubMed, Embase, CENTRAL, and Web of Science) and four Chinese databases (SinoMed, VIP information database, China National Knowledge Infrastructure, and Wanfang Data Information Site) without no limitation on language or areas (The strategy was described in Supplementary Table S3). Furthermore, we searched ClinicalTrials.gov and the Chinese Clinical Trial Registry, and tracked citations on relevant systematic reviews.

### 2.2 Inclusion criteria

#### 2.2.1 Types of participants

Patients with CHD diagnosed by a clinician or according to recognized diagnostic criteria. There were no restrictions on age, gender, race, or CHD type.

#### 2.2.2 Types of interventions

Participants in the control group were treated with conventional treatment (anticoagulation, antiplatelet aggregation, receptor blockers, nitrates, angiotensin-converting enzyme inhibitors, statins, etc.). Participants in the experimental group are provided with SXNI in addition to the conventional treatment.

#### 2.2.3 Outcome measures

The primary outcomes assessed in this study were the clinical efficacy (The criteria was demonstrated in Supplementary Table S4) and angina symptoms (duration of angina and frequency of angina attack). Furthermore, we collected other valuable outcomes that can obtain the accurate data. 1) Cardiac function: cardiac index (CI), cardiac output (CO), left ventricular ejection fraction (LVEF), and left ventricular end-diastolic dimension (LVEDD); 2) electrocardiogram (ECG) efficacy; 3) thrombosis factors: fibrinogen (FIB), plasma viscosity (PV), whole blood high shear rate (HS) and hematocrit (HCT); 4) blood lipid index: total cholesterol (TC), triglyceride (TG), low-density lipoprotein (LDL), and high-density lipoprotein (HDL); 5) others: C-reactive protein (CRP), brain natriuretic peptide (BNP); and 6) adverse reactions. The included studies had at least one of these outcomes.

#### 2.2.4 Types of studies

Randomized control trials (RCTs).

### 2.3 Exclusion criteria

We excluded the following studies: 1) Case reports, animal studies, case reports, and meta-analysis, etc.,; 2) studies treated with other traditional chinese medicine such as Chinese botanical drug, massage, scraping, cupping; 3) studies with incomplete or inadequate data; 4) studies involving patients with other serious diseases such as severe liver, brain, and kidney diseases, etc.

### 2.4 Literature screening and data extraction

Two researchers (SML and STY) independently conducted the study screening and data extraction according to the inclusion and exclusion criteria. EndNote X9 was used to manage the records and removed duplicates. The extracted data included 1) sample characteristics: authors, publication year, sample size, mean age of participants, types of CHD, and the injection method of SXNI; 2) study design: randomization, allocation concealment, and blinding; 3) information of outcomes: clinical efficacy rate, angina symptoms, and other valuable outcomes. A third researcher (JZC) reviewed and confirmed the final data extraction sheets. For missing data, efforts were made to contact the authors of the respective articles. If unsuccessful, the study had to be excluded from the analysis.

### 2.5 Evaluation of risk of bias

Two researchers (SML and STY) assessed the quality of included studies using the Cochrane risk of bias tool 2.0 (RoB2.0), in which six domains were evaluated: 1) randomization process, 2) deviations from the intended interventions, 3) missing outcome data, 4) measurement of the outcome, 5) selection of the reported outcome, and 6) overall bias. Any discrepancies that arose during the assessment were resolved through discussion with a third researcher (JZC). Each entry was either rated as low, high, or unclear.

### 2.6 Data synthesis and analysis

Statistical analyses were performed using Review Manager version 5.4 (Cochrane Collaboration, Copenhagen, Denmark) and STATA 15.1 (StataCorp, College Station, TX, United States). For dichotomous data, the relative risk (RR) was calculated, while for continuous variables, the mean difference (MD) was calculated. Confidence intervals (CIs) were set at 95%, and a *p* < 0.05 indicated statistical significance for the overall effect. Heterogeneity among studies was quantified using the inconsistency index (I2). If I2 < 50% and *p* > 0.05, the fixed-effects model was used to pool the data; otherwise, the random-effects model was used. The mean and standard deviation for variable changes before and after the treatment are calculated with the methods provided in Chapter 16.1.3.2 of Cochrane Handbook Version 5.0.2 (Corr = 0.40).

### 2.7 Sensitivity, subgroup and meta-regression analysis

Preplanned subgroup analyses were conducted to investigate the potential influence of specific characteristics (duration of treatment, dose of SXNI, types of CHD, and the mode of administration) on the efficacy of SXNI. Furthermore, univariate meta-regression analyses using sample size, mean age, and year of publication as co-variables were performed to identify the confounding factors that may result in heterogeneity. Sensitivity analysis was performed by excluding studies with a high degree of overall bias or by excluding single study to assess the stability of the results and query the sources of heterogeneity.

### 2.8 Publication bias

When the number of studies exceeded 10, we assessed the possibility of publication bias using funnel plots and Egger’s regression test. In addition, we used the trim-and-fill analysis to estimate the number of missing studies through iterative methods, and assess the effect of publication bias on the interpretation of the results.

### 2.9 Quality of evidence

We used Grading of Recommendation, Assessment, Development, and Evaluation (GRADE) to assess the quality of evidence for each outcome, in which five domains were evaluated: 1) study limitations were assessed according to RoB2.0; 2) consistency was evaluated using I2 values and the agreement of 95% confidence; 3) indirectness; 4) precision was examined by the optimal information sample size; and 5) publication bias and the number of included studies (Gonzalez-Padilla and Dahm, 2021). Similarly, the quality of evidence by GRADE was also decided with consensus.

### 2.10 Evaluation of this SR

After the completion of this study, two researchers (FCN and ZCY), who had no conflict of interest with the study, assessed the methodological quality and risk of bias using the Modified Quality Assessment Scale for Systematic Reviews (AMSTAR-2) (Shea et al., 2017) and Risk of Bias in Systematic Reviews (ROBIS) tool (Whiting et al., 2016). In case of any disagreements, they resolved them through discussion. If necessary, the opinion of a third expert (HHY) was sought to provide further insights. Furthermore, we will refine this meta-analysis according to the results of reviewing until all entries were satisfied.

## 3 Results

### 3.1 Results of study selection

**FIGURE 1:**
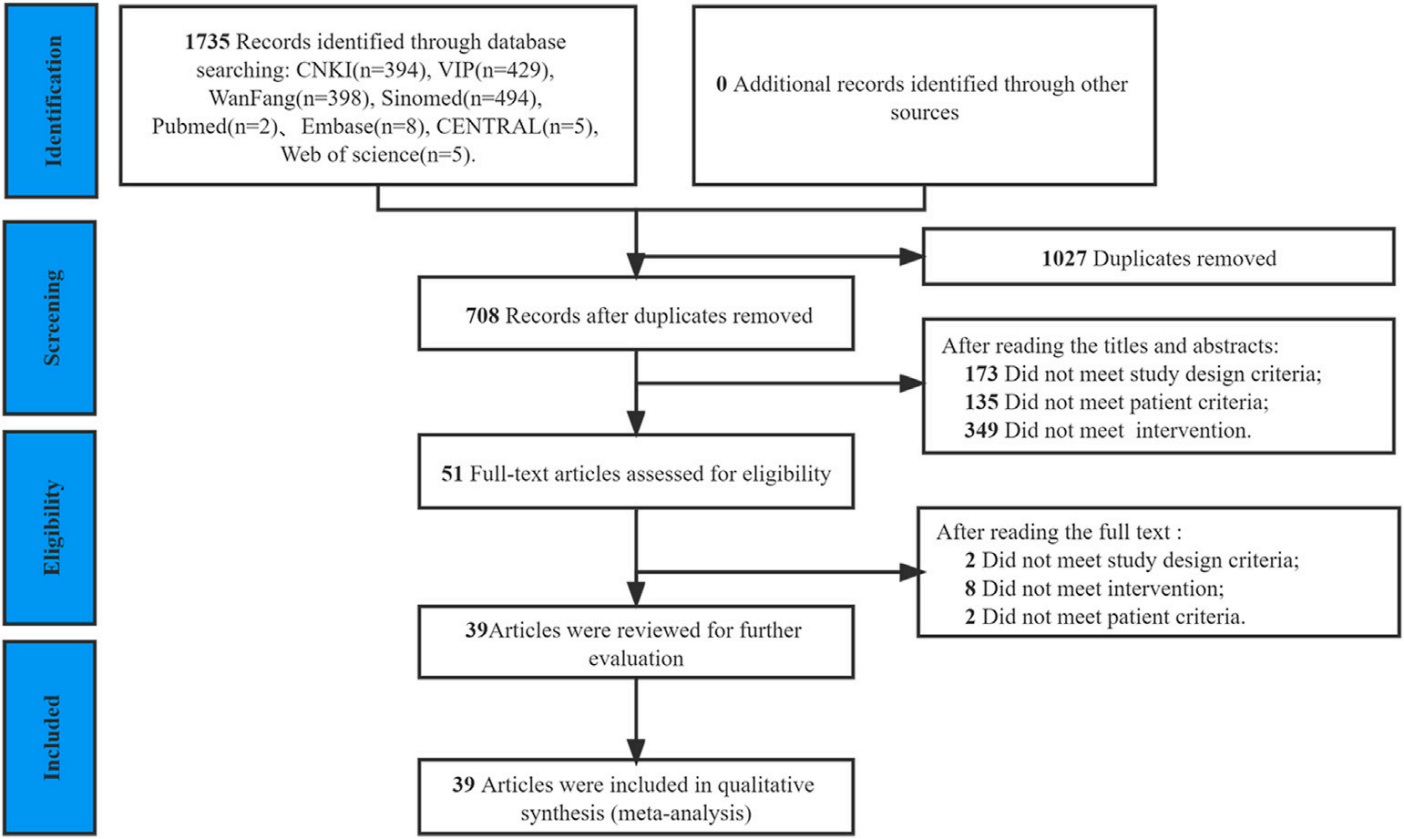
PRISMA flow diagram of study selection process.

A total of 1,735 studies were initially searched. Among them, 1,027 were duplicates, and 657 were excluded after reading the title or abstract. Finally, 51 trials were reviewed at the full-text level for further evaluation, of which 12 were excluded (Reasons of excluding studies were provided in Supplementary Table S5), and the remaining 39 studies (Cui et al., 2008; Gao et al., 2009; Wang and Zhang, 2009; Chai, 2010; SUn, 2010; Zhong, 2010; Zhou, 2010; Zhuang and Wu, 2010; Gao and Feng, 2011; Sui, 2011; Yang, 2011; Zhang and Yu, 2011; Zhao and Liu, 2011; Ma and Xi, 2012; Meng et al., 2012; Xu, 2012; Zhang, 2012; Liu and Zhang, 2013; Yuan, 2013; Dai, 2014; He et al., 2014; Ren and Wang, 2014; Zhu, 2014; Li, 2015; Liu and Cheng, 2015; Liu et al., 2015; Wang, 2015; Chen et al., 2016; Wu, 2016; Zhang, 2016; Zhou, 2017; Liu, 2018; Zhang et al., 2018; Sun et al., 2019; Cai, 2021; Wang, 2021; Wang, 2021; He et al., 2022; Zhang, 2022) were included in the final review (Figure 1).

### 3.2 Characteristics of the included studies

A total of 39 RCTs involving 3,779 patients with CHD were included. All trials were conducted in China from 2008 to 2022. There was a total of 1,920 participants in the experimental groups and 1,859 in the control groups. The average age of participants ranged from 48 to 72 years old. Regarding disease subtypes, one trial only recruited patients with stable angina pectoris (SAP) (Zhang, 2012), and nineteen studies recruited patients with unstable angina pectoris (UAP) (Chai, 2010; Zhou, 2010; Gao and Feng, 2011; Sui, 2011; Yang, 2011; Ma and Xi, 2012; Liu and Zhang, 2013; Yuan, 2013; He et al., 2014; Zhu, 2014; Li, 2015; Liu and Cheng, 2015; Liu et al., 2015; Wang, 2015; Wu, 2016; Liu, 2018; Sun et al., 2019; Wang, 2021; Zhang, 2022). The other studies did not specify the type of CHD. All the studies employed a two-arm design, with SXNI combination therapy as the experimental group and conventional therapy as the control group. In experimental groups, the majority of studies used the intravenous route as the mode of administration for SXNI, and only two studies (Sun et al., 2019; Zhang, 2022) used intramuscular injection; the daily dosage of SXNI ranged from 3 mL to 30 mL, and the duration of treatment spanned from 1week to 1 month. In the control groups, conventional therapy, including anticoagulants, antiplatelets, and receptor blockers, was used. The characteristics of the included studies were indicated in Table 1.

**TABLE 1:** Detailed information about the studies included.

Study	Subtypes of disease	Sample		Gender (M/F)		Age distribution		Treatment	Administration	Duration	Outcomes
		T	C	T	C	T	C				
Zhang (2012)	SAP	27	27	18/9	17/10	54.3	55.6	SXNI 20mL/qd + CT/CT	ivgtt	3 weeks	1, 9,10,15,16,18
Zhang. (2022)	UAP	30	30	18/12	16/14	48.75 ± 18.63	48.87 ± 18.3	SXNI 10mL/qd + CT/CT	im	2 weeks	1,4,5,6,7,11,12,13,14,19
Sun et al. (2019)	UAP	45	44	22/23	20/24	51.67 ± 4.83	52.49 ± 5.15	SXNI 3mL/bid + CT/CT	im	2 weeks	1,9,19
Zhang et al. (2018)	CHD	38	38	23/15	24/14	61.37 ± 4.21	60.83 ± 4.59	SXNI 15mL/qd + CT/CT	ivgtt	2 weeks	2,3,4,5,6
Zhang and Yu. (2011)	CHD	30	30	NR	NR	NR	NR	SXNI 20mL/qd + CT/CT	ivgtt	4 weeks	1,10,17,18,19
Ma and Xi. (2012)	UAP	53	49	37/16	30/19	68.79 ± 10.31	68.12 ± 10.27	SXNI 20mL/qd + CT/CT	ivgtt	10 days	9,10,15,17,19
Zhuang and Wu.(2010)	CHD	129	109	71/58	67/42	58.4 ± 7.5	57.8 ± 8.2	SXNI 20mL/qd + CT/CT	ivgtt	2 weeks	1,10,15,16,17
Wu. (2016)	UAP	40	40	24/16	25/15	61.2 ± 3.1	61.5 ± 3.2	SXNI 20mL/qd + CT/CT	ivgtt	2 weeks	8,10,19
Wang. (2021)	UAP	65	65	35/30	34/31	62.05 ± 6.59	61.59 ± 6.28	SXNI 20mL/qd + CT/CT	ivgtt	15 days	2,3,9,11,12,13,18,19
Wang. (2021)	AP	50	50	27/23	26/24	65.34 ± 3.55	65.29 ± 3.88	SXNI 20mL/qd + CT/CT	ivgtt	1 week	1,2,16
Liu et al. (2015)	UAP	42	42	24/18	25/17	60.5 ± 3.2	61.3 ± 3.4	SXNI 20mL/qd + CT/CT	ivgtt	2 weeks	1,2,3,8,10,19
Chen et al. (2016)	AP	48	48	25/23	23/25	52.4 ± 6.5	53.1 ± 5.6	SXNI 20mL/qd + CT/CT	ivgtt	2 weeks	1,9,15,16,17,18,19
He et al. (2014)	UAP	62	60	32/30	31/29	55.80 ± 3.14	52.80 ± 2.90	SXNI 20mL/qd + CT/CT	ivgtt	4 weeks	1,9,10,11,12,13,14,16,17,19
Dai. (2014)	AP	51	50	25/26	29/21	68.5 ± 10.1	69.1 ± 10.4	SXNI 20mL/qd + CT/CT	ivgtt	2 weeks	3,11,12,13,14,15,16
Chai. (2010)	UAP	58	42	34/24	30/12	64.3 ± 2.3	62.6 ± 1.9	SXNI 20mL/qd + CT/CT	ivgtt	1 week	1,9,11,12,13,14,15,17,19
Cai. (2021)	AP	50	50	27/23	25/25	50.46 ± 7.84	51.12 ± 8.72	SXNI 20mL/qd + CT/CT	ivgtt	4 weeks	1,9,10,11,12,19
Zhu. (2014)	UAP	48	48	26/22	30/18	63.8 ± 11.2	64.1 ± 10.8	SXNI 20mL/qd + CT/CT	ivgtt	2 weeks	1,9,10
Zhou. (2010)	UAP	100	100	NR	NR	NR	NR	SXNI 10mL/bid + CT/CT	ivgtt	NR	1,16,19
Zhou. (2017)	AP	30	30	NR	NR	NR	NR	SXNI 20mL/qd + CT/CT	ivgtt	1 month	1,2,3
Zhong. (2010)	AP	90	90	53/37	51/39	59.5 ± 11.5	61.3 ± 10.5	SXNI 20mL/qd + CT/CT	ivgtt	4 weeks	1,9,15,16,18,19
Zhang. (2016)	CHD	50	50	26/24	25/25	66.9 ± 6.7	66.5 ± 6.4	SXNI 30mL/qd + CT/CT	ivgtt	NR	9,11,12
Wang and Zhang. (2009)	AP	46	42	30/16	33/9	54 ± 8	54 ± 7	SXNI 20mL/qd + CT/CT	ivgtt	2 weeks	1,9,15,16,19
Li et al. (2015)	UAP	36	36	20/16	19/17	64.3 ± 1.2	63.1 ± 1.3	SXNI 25mL/qd + CT/CT	ivgtt	4 weeks	1,9,10
He et al. (2022)	CHD	30	30	NR	NR	57.28 ± 4.19	57.64 ± 4.57	SXNI 20mL/qd + CT/CT	ivgtt	3 days	1,4,5,19
Liu and Zhang. (2013)	UAP	50	50	27/23	26/24	58.7 ± 6.5	57.9 ± 5.3	SXNI 20mL/qd + CT/CT	ivgtt	NR	1,8,10
Yuan. (2013)	UAP	24	24	NR	NR	NR	NR	SXNI 20mL/qd + CT/CT	ivgtt	2 weeks	15,16,17
Yang. (2011)	UAP	60	60	NR	NR	NR	NR	SXNI 10mL/qd + CT/CT	ivgtt	15 days	1,9,16,19
Meng et al. (2012)	AP	42	42	28/14	30/12	72.4 ± 8.9	71.2 ± 9.6	SXNI 20mL/qd + CT/CT	ivgtt	20 days	1,9,10,16
Xu. (2012)	AP	25	25	14/11	13/12	56.1 ± 7.2	55.6 ± 6.6	SXNI 20mL/qd + CT/CT	ivgtt	10days	2,3,19
Ren and Wang. (2014)	AP	40	40	21/19	23/17	57	58	SXNI 20mL/qd + CT/CT	ivgtt	2 weeks	1,9,15,16,19
Wang. (2015)	UAP	52	52	28/24	31/21	54.2 ± 2.1	56.2 ± 2.6	SXNI 20mL/qd + CT/CT	ivgtt	4 weeks	1,10,11,12
Sun. (2010)	AP	42	38	30/12	28/10	NR	NR	SXNI 20mL/qd + CT/CT	ivgtt	10 days	1,9,11,19
Sui. (2011)	UAP	30	30	NR	NR	NR	NR	SXNI 20mL/qd + CT/CT	ivgtt	2 weeks	1,2,3,10
Zhao and Liu. (2011)	AP	26	24	15/11	14/10	60	63	SXNI 20mL/qd + CT/CT	ivgtt	2 weeks	1,2,3,19
Liu. (2018)	UAP	40	40	23/17	23/17	58.90 ± 4.72	58.76 ± 4.50	SXNI 20mL/qd + CT/CT	ivgtt	2 weeks	2,3,8,10
Gao et al. (2009)	AP	100	100	NR	NR	NR	NR	SXNI 20mL/qd + CT/CT	ivgtt	2 weeks	1,9,15,16
Cui et al. (2008)	AP	60	50	32/28	27/23	62.8	56.6	SXNI 20mL/qd + CT/CT	ivgtt	15 days	1,9,15,16,18
Gao and Feng. (2011)	UAP	33	36	19/14	23/13	NR	NR	SXNI 20mL/qd + CT/CT	ivgtt	2 weeks	1,2,3,19
Liu et al. (2015)	UAP	48	48	25/23	32/16	59.38 ± 6.22	54.87 ± 4.95	SXNI 20mL/qd + CT/CT	ivgtt	2 weeks	1,9,10

Abbreviations: AP, angina pectoris; C, control group; CHD, coronary heart disease; CT, conventional therapy; NR, not reported; SAP, stable angina pectoris; SXNI, Shuxuening injection; T, treatment group; UAP, unstable angina pectoris; im, intramuscular injection; ivgtt, intravenous injection; Outcomes 1:clinical efficacy; 2:angina duration; 3: angina attack frequency; 4: Left Ventricular Ejection Fractions (LVEF); 5: left ventricular end-diastolic dimension (LVEDD); 6: cardiac index; 7: cardiac output; 8: BNP; 9: ECG, efficacy; 10: CRP; 11: total cholesterol (TC); 12: triglyceride (TG); 13: low-density lipoprotein (LDL); 14: high-density lipoprotein (HDL); 15: Fibrinogen (FIB); 16: plasma viscosity (PV); 17: whole blood high shear rate(HS); 18: hematocrit (HCT); 19:adverse reaction.

### 3.3 Risk of bias

**FIGURE 2:**
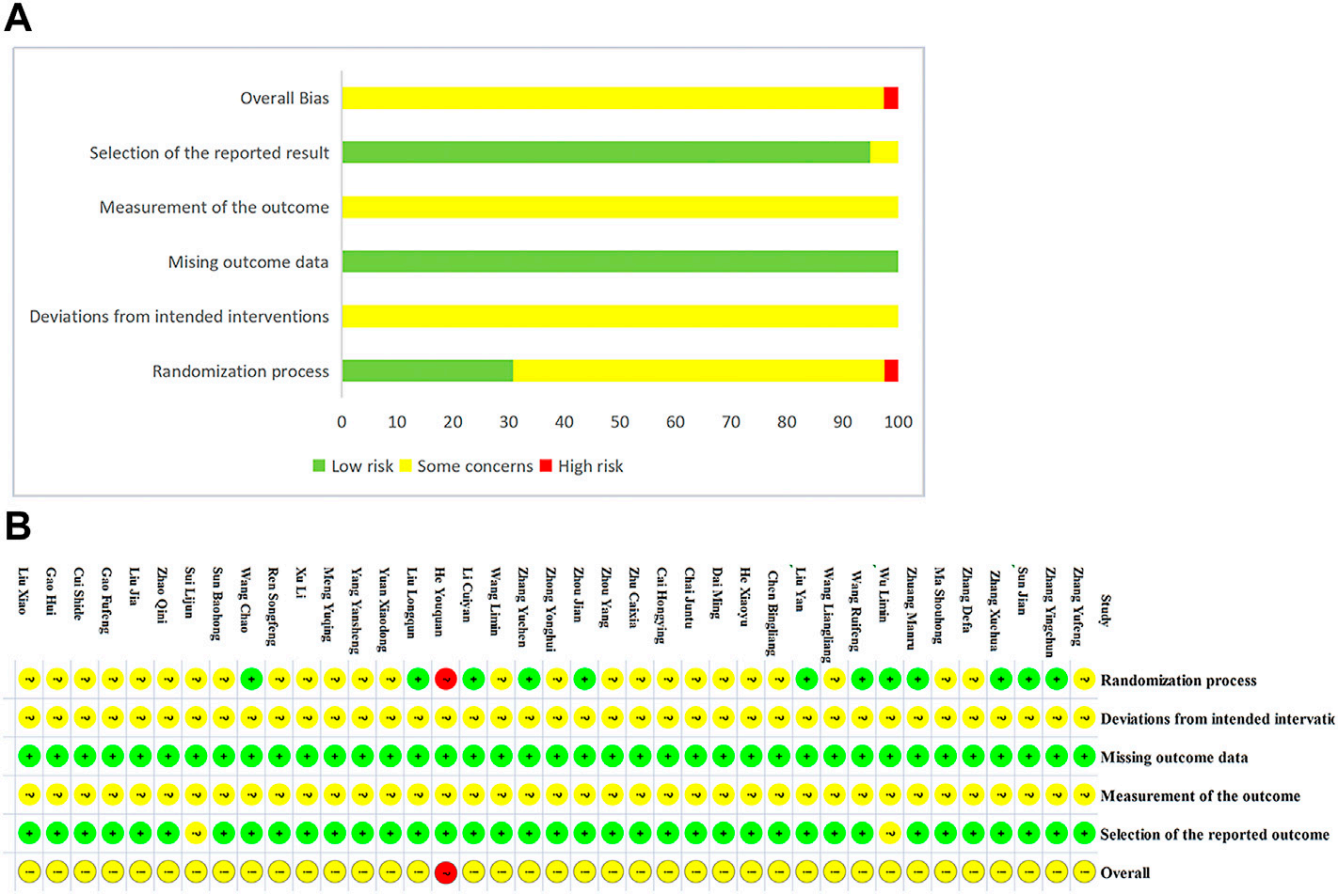
Risk of bias. **(A)** Risk of bias summary. **(B)** Risk of bias graph.

Twelve studies (Zhuang and Wu, 2010; Liu and Zhang, 2013; Li, 2015; Liu et al., 2015; Wang, 2015; Wu, 2016; Zhang, 2016; Zhou, 2017; Zhang et al., 2018; Sun et al., 2019; Wang, 2021; Zhang, 2022) provided a sufficient randomization process to generate random sequences with a low risk of bias; while one RCT (He et al., 2022) was assessed as high risk due to the possibility of patients being grouped based on physician preferences. The remaining studies did not provide specific details of randomization, and therefore we evaluated the risk of bias as unclear. Additionally, none of the studies provided information on allocation concealment, blinding of participants, and outcome assessment, so we evaluated the risk of bias as unclear. All studies included in the analysis published complete data regarding the outcomes, leading us to rate the risk of bias as low. We were concerned about the selection of the reported results from two studies (Sui, 2011; Wu, 2016), and as a result, we evaluated the risk of bias as unclear. Overall, one study had a domain rated as having an extreme risk of bias, all studies had at least one domain rated as having an uncertain risk of bias due to a lack of information (Figure 2).

### 3.4 Primary outcomes

#### 3.4.1 Clinical efficacy

**FIGURE 3:**
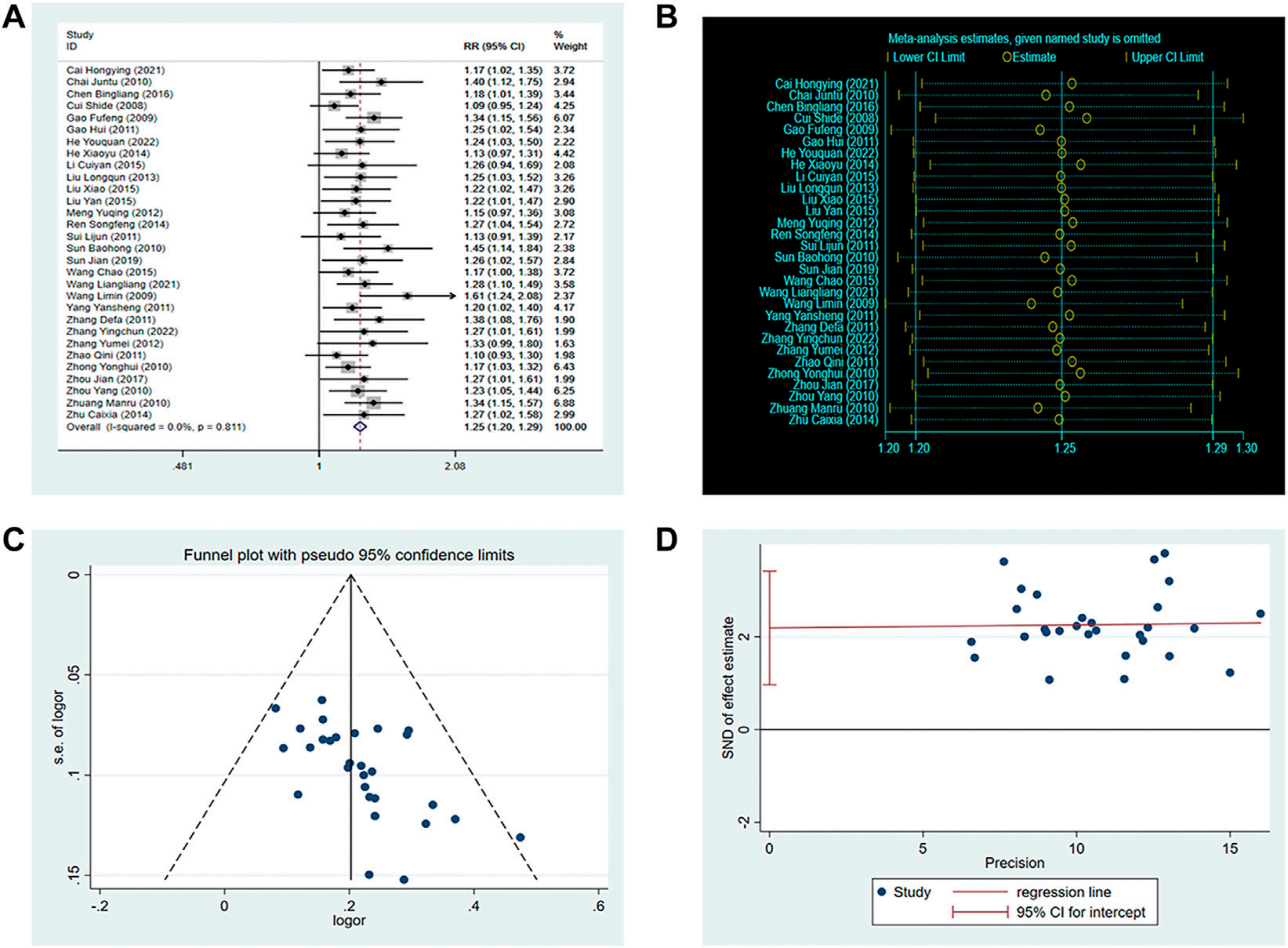
Effect of SXNI on clinical efficacy. **(A)** Forest plot of clinical efficacy. **(B)** Sensitivity analysis revealed the reliability of conclusion. **(C)** Funnel plots revealed the publication bias. **(D)** Egger’s test quantified the publication bias.

A total of thirty RCTs involving 3,012 patients reported clinical efficacy. The fixed-effects model was used for the meta-analysis because of the low heterogeneity among the studies (*p* = 0.81, I2 = 0%). The results demonstrated that the efficacy of SXNI combination therapy was superior to conventional treatment (OR: 1.25, 95%CI: 1.20 to 1.29, *p* < 0.00001, Figure 3A). A sensitivity analysis was performed, and the result showed that the findings of the meta-analysis were not altered, suggesting that the conclusion was reliable (Figure 3B). Furthermore, the asymmetry of the funnel plot and the result of the Egger’s test (*p* = 0.001) indicated the presence of potential publication bias (Figures 3C,D). Therefore, we conducted a trim-and-fill test to assess the effect of tghe publication bias on the interpretation of the results. The result indicated that this publication bias did not affect the estimates, although several RCTs showing negative findings remained unpublished (Supplementary Table S6).

**FIGURE 4:**
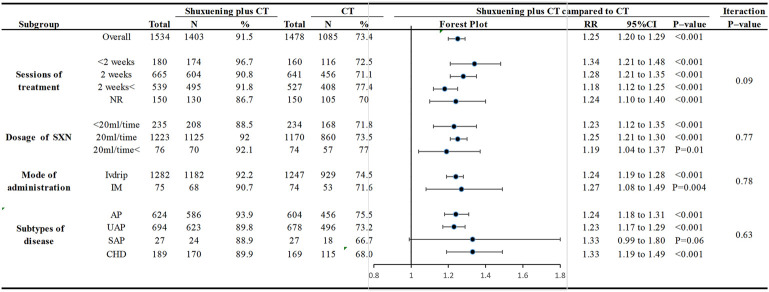
Subgroup analysis of the clinical efficacy.

Furthermore, we conducted subgroup analyses based on the duration of treatment, dose of SXNI, mode of administration, and subtypes of disease to determine the effects of these characteristics on the efficacy of SXNI (Figure 4). The results indicated that most subgroups were consistent with the overall findings, suggesting that the duration of treatment, dose of SXNI, and mode of administration did not significantly affect the positive effect of SXNI in treating CHD. However, when considering the subtypes of disease, SXNI combination therapy did not show a significant positive effect in treating SAP (OR: 1.33, 95%CI: 0.99 to 1.80, *p* = 0.06).

#### 3.4.2 Improvement of angina symptoms

**FIGURE 5:**
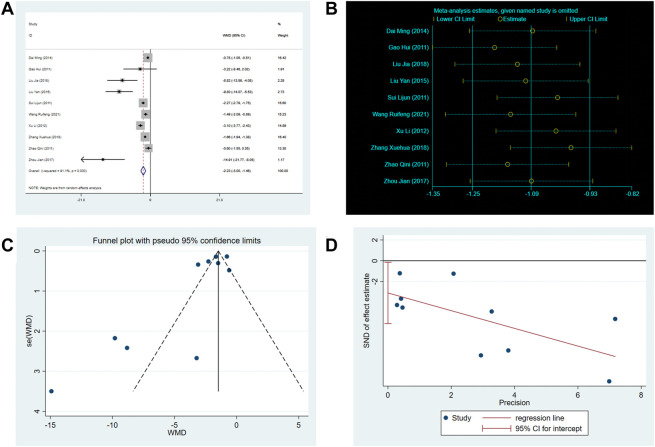
Effect of SXNI on frequency of angina attack. **(A)** Forest plot of angina frequency. **(B)** Sensitivity analysis revealed the reliability of conclusion. **(C)** Funnel plots revealed the publication bias. **(D)** Egger’s test quantified the publication bias.

There are 10 RCTs involving 760 patients reported the frequency of angina attacks. The random-effects model was used because of the high heterogeneity (*p* < 0.0001, I2 = 91%). As shown in Figure 5, the SXNI combination therapy resulted in a greater reduction in the frequency of angina attacks compared to conventional treatment (MD: –2.23, 95%CI: –3.00 to −1.46, *p* < 0.00001, Figure 5A). The asymmetry of the funnel plot and the result of the Egger’s test (*p* = 0.041) revealed a publication bias (Figures 5C,D). However, the trim-and-fill analysis showed that the publication bias did not affect the conclusion.

**FIGURE 6:**
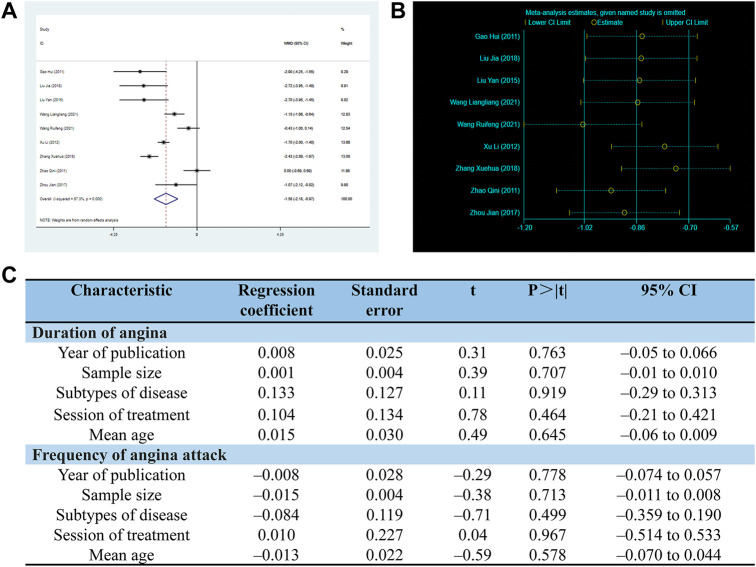
Effect of SXNI on angina symptoms. **(A)** Forest plot of angina duration. **(B)** Sensitivity analysis revealed the reliability of conclusion. **(C)** Meta-regression was used to find the sources of heterogeneity.

There are 9 RCTs involving 699 patients reported the duration of angina. The random-effects model was used due to the high heterogeneity (*p* < 0.0001, I2 = 87%). As shown in Figure 6, the SXNI combination therapy resulted in a greater reduction in angina duration compared to conventional treatment (MD: –1.58, 95%CI: –2.18 to −0.97, *p* < 0.00001, Figure 6A).

Considering the high heterogeneity observed in both angina duration and frequency, the meta-regression was conducted using sample size, mean age, subtypes of disease, duration of treatment, and year of publication as co-variables to investigate the sources of heterogeneity. However, the analysis did not reveal any linear relationships between the variables and the outcome indicators, indicating that these variables were not the sources of heterogeneity (Figure 6C). Sensitivity analysis was also performed, and the results demonstrated the reliability of SXNI combination therapy in improving angina symptoms (Figure 5B, Figure 6B).

### 3.5 Secondary outcome

#### 3.5.1 Improvement of cardiac function

Improvements in cardiac function were assessed using LVEF, LVEDD, cardiac index (CI), and cardiac output (CO). One RCT (Zhang, 2022) reported the cardiac output, which revealed that SXNI combination therapy was superior to conventional therapy in improving CO.

Two RCTs reported the CI. Since there was low heterogeneity (*p* = 0.74, I2 = 0%) observed, the fixed-effects model was utilized. The meta-analysis found that SXNI combination therapy was superior to conventional therapy in improving CI (MD: 0.31, 95%CI: 0.16 to 0.45, *p* < 0.00001, Supplementary Figure S2A).

Three RCTs reported LVEF and LVEDD. The random-effects model was used because of the high heterogeneity in LVEF (*p* = 0.13, I2 = 51%) and LVEDD (*p* = 0.006, I2 = 80%). The meta-analysis revealed that SXNI combination therapy resulted in a significant increase in LVEF (MD: 8.17, 95%CI: 5.74 to 10.60, *p* < 0.00001, Supplementary Figure S2B) and a significant decrease in LVEDD (MD: –5.12, 95%CI: –5.95 to −4.28, *p* < 0.00001, Supplementary Figure S2D) compared to conventional treatment. Sensitivity analysis revealed that the heterogeneity of LVEF (*p* = 0.64, I2 = 0%) and LVEDD (*p* = 0.41, I2 = 0%) significantly reduced when one RCT (He et al., 2022) was excluded (Supplementary Figure S2C,E). Different from other studies, the overall bias in this study was high which may contributed to the methodological heterogeneity.

#### 3.5.2 Improvement of ECG efficacy

A total of 20 RCTs involving 2,099 patients reported the ECG efficacy, which depends on the electrocardiogram improvement of patient. The fixed-effects model was used because of the low heterogeneity (*p* = 0.35, I2 = 8.6%). The meta-analysis found that the SXNI combination therapy was superior to conventional treatment in improving ECG efficacy (OR: 1.35, 95%CI: 1.27 to 1.42, *p* < 0.00001, Supplementary Figure S3A). Sensitivity analysis revealed the robustness and reliability of conclusion (Supplementary Figure S3B). Furthermore, the asymmetry of the funnel plot and the result of the Egger’s test (*p* = 0.001) revealed a publication bias (Supplementary Figure S3C,E). The trim-and-fill analysis was conducted and suggested this publication bias did not affect the conclusion (Supplementary Table S7).

#### 3.5.3 Improvement of CRP

CRP, one of the important predictors of cardiovascular events, is closely related to the occurrence of atherosclerosis (Tajfard et al., 2019). Fifteen RCTs involving 1,432 patients reported the CRP levels. The random-effects model was used because of the high heterogeneity (*p* < 0.00001, I2 = 90%). The meta-analysis found that SXNI combination therapy was superior to conventional therapy in decreasing CRP (MD: –2.06, 95%CI: –2.58 to −1.54, *p* < 0.00001, Supplementary Figure S5A). The funnel plot and result of the Egger’s test (*p* = 0.335) revealed that there was no publication bias (Supplementary Figure S5C,D). Additionally, the meta-regression found that sample size, mean age, disease subtypes, duration of treatment, and year of publication were not sources of heterogeneity (Supplementary Table S8). The sensitivity analysis also indicated the reliability of the conclusion (Supplementary Figure S5B).

#### 3.5.4 Improvement of BNP

Four RCTs reported BNP levels. The fixed-effects model was used because of the low heterogeneity (*p* = 0.97, I2 = 0%). The result found that SXNI combination therapy was superior to conventional therapy in decreasing BNP (MD: –21.81, 95%CI: –27.86 to −15.77, *p* < 0.00001, Supplementary Figure S6A). Sensitivity analysis revealed the reliability of the conclusion (Supplementary Figure S6B).

#### 3.5.5 Improvement of blood lipid

Dyslipidemia, which can lead to insufficient blood perfusion in the heart, is one of the main causes of CHD (Ma et al., 2023). Therefore, the total cholesterol (TC), triglyceride (TG), low-density lipoprotein (LDL), and high-density lipoprotein (HDL) were used in assessing blood lipid profiles.

Nine RCTs involving 897 patients reported TC levels. The random-effects model was used due to the heterogeneity (*p* < 0.0001, I2 = 95%). The meta-analysis revealed that SXNI combination therapy was superior to conventional therapy in decreasing TC (MD: –1.02, 95%CI: –1.48 to −0.55, *p* < 0.00001, Supplementary Figure S7A). Sensitivity analysis revealed the reliability of the conclusion (Supplementary Figure S7B).

Eight RCTs involving 817 patients reported TG levels. The random-effects model was used because of the heterogeneity (*p* < 0.0001, I2 = 90%). The meta-analysis revealed that SXNI combination therapy was superior to conventional therapy in decreasing TG (MD: –0.81, 95%CI: –1.06 to −0.57, *p* < 0.00001, Supplementary Figure S8A). Sensitivity analysis confirmed the robustness of the conclusion (Supplementary Figure S8B).

Six RCTs involving 613 patients reported the LDL levels. The random-effects was used because of the heterogeneity (*p* < 0.0001, I2 = 92%). The meta-analysis revealed that SXNI combination therapy was superior to conventional therapy in decreasing LDL (MD: –1.12, 95%CI: –1.48 to −0.75, *p* < 0.00001, Supplementary Figure S9A). Sensitivity analysis confirmed the robustness of the conclusion (Supplementary Figure S9B).

Four RCTs involving 383 patients reported the HDL levels. The random-effects model was used because of the heterogeneity (*p* = 0.0001, I2 = 86%). The meta-analysis revealed that SXNI combination therapy was superior to conventional therapy in increasing HDL (MD: 0.68, 95%CI: 0.54 to 0.83, *p* < 0.00001, Supplementary Figure S10A). The sensitivity analysis revealed that the heterogeneity (*p* = 0.28, I2 = 21%) declined when one RCT (Chai, 2010) was excluded. Different from other studies, the duration of treatment in this study was less than 2 weeks, which may lead to the clinical heterogeneity (Supplementary Figure S10B).

Considering the high heterogeneity of TC, TG and LDL, we performed a meta-regression using sample size, mean age, subtypes of disease, duration of treatment, and year of publication as co-variables to query the sources of heterogeneity. However, there were no linear relationships between the variables and the outcome index, indicating that they were not sources of heterogeneity (Supplementary Table S9).

#### 3.5.6 Improvement of thrombosis factors

Fibrinogen (FIB), plasma viscosity (PV), whole blood high shear rate(HS), and hematocrit (HCT) were used to assess the improvement of thrombosis factors.

The level of FIB is associated with the risk of CHD and the severity of atherosclerosis. Increased levels of FIB have been established as an indicator of coronary risk (Danesh et al., 1998). Twelve RCTs involving 1,397 patients reported FIB levels. The random-effects model was used because of the high heterogeneity (*p* < 0.0001, I2 = 98%). The meta-analysis results revealed that SXNI combination therapy was superior to conventional therapy in decreasing FIB (MD: –1.08, 95%CI: –1.21 to −0.94, *p* < 0.00001, Supplementary Figure S11A). The asymmetry of the funnel plot and the result of the Egger’s test (*p* = 0.001) indicated a potential publication bias (Supplementary Figure S11C,D). The trim-and-fill analysis revealed that several RCTs showing negative findings remained unpublished (Supplementary Table S10), which could affect the conclusion. However, sensitivity analysis confirmed the robustness of the existing conclusion (Supplementary Figure S11B). Furthermore, meta-regression was conducted and not found the source of heterogeneity (Supplementary Table S11).

A meta-analysis involving 28,605 patients showed that PV was strongly associated with the risks of CHD and all-cause mortality (Lowe et al., 2023). Fifteen RCTs involving 1,821 patients reported PV levels. The random-effects model was used because of the high heterogeneity (*p* < 0.0001, I2 = 97%). The meta-analysis revealed that SXNI combination therapy was superior to conventional therapy in decreasing PV (MD: –0.37, 95%CI: –0.49 to −0.25, *p* < 0.00001, Supplementary Figure S12A). The funnel plot and the result of the Egger’s test (*p* = 0.08) revealed that there were no publication bias (Supplementary Figure S12C,D). Sensitivity analysis confirmed the reliability of the conclusion (Supplementary Figure S12B). Furthermore, the meta-regression found the mean age of patients may be the source of heterogeneity (Supplementary Table S11). Subgroup analyses showed that whether mean age <60 (MD: –0.19, 95%CI: –0.30 to −0.07, *p* = 0.002), mean age ≥60 (MD: –0.34, 95%CI: –0.60 to −0.07, *p* = 0.01) or no mentioned (MD: –0.78, 95%CI: –1.03 to −0.52, *p* < 0.00001) all decreased PV better in SXNI combination therapy (Supplementary Figure S13).

Results based on the Framingham study showed a strong association between HCT and CHD morbidity and mortality (Gagnon et al., 1994). Six RCTs involving 600 patients reported HCT levels. The random-effects model was used because of the high heterogeneity (*p* < 0.0001, I2 = 100%). The meta-analysis revealed that SXNI combination therapy was superior to conventional therapy in decreasing HCT (MD: –4.72, 95%CI: –6.78 to −2.66, *p* < 0.00001, Supplementary Figure S14A). Sensitivity analysis confirmed the reliability of conclusion (Supplementary Figure S14B). Furthermore, the meta-regression did not find any source of heterogeneity (Supplementary Table S11).

Six RCTs involving 718 patients reported HS levels. The random-effects model was used because of the high heterogeneity (*p* < 0.0001, I2 = 88%). The meta-analysis results revealed that SXNI combination therapy was superior to conventional therapy in decreasing HS (MD: –0.90, 95%CI: –1.35 to −0.46, *p* < 0.00001, Supplementary Figure S15A). Sensitivity analysis confirmed the reliability of conclusion (Supplementary Figure S15B). Furthermore, meta-regression was conducted and not found the source of heterogeneity (Supplementary Table S11).

#### 3.5.7 Adverse reactions

Due to variations in the criteria for reporting adverse reactions across the included studies, we only performed a descriptive analysis. A total of 19 out of the included 39 studies reported adverse reactions. Of these, no adverse reactions were observed in eight studies (Danesh et al., 1998; Wang and Zhang, 2009; Zhou, 2010; Yang, 2011; Zhang and Yu, 2011; Zhao and Liu, 2011; Xu, 2012; Ren and Wang, 2014; He et al., 2022). Two studies (SUn, 2010; He et al., 2014) reported no adverse reactions in the experimental group, but adverse reactions such as facial flushing and the slightly increased of alanine aminotransferase (ALT) were observed in the control group. The main adverse reactions in five studies (Zhong, 2010; Ma and Xi, 2012; Li, 2015; Sun et al., 2019; Zhang, 2022) were dizziness and nausea. Four studies (Chai, 2010; Zhong, 2010; Cai, 2021; Wang, 2021) also reported other adverse reactions such as rashes and gastrointestinal discomfort, but there was no significant difference between groups. Overall, the SXNI combination therapy did not increase the occurrence of adverse reactions. (The details of adverse reactions were described in Supplementary Table S12).

### 3.6 Quality of evidence

The GRADE approach was used to systematically assess the quality of 17 outcome indicators (Table 2). The results revealed moderate-quality evidence for clinical efficacy, angina duration, CRP, EGG changes, TC, TG, LDL, PV, HS, and HCT. In addition, low-quality evidence was indicated for the angina attack frequency, BNP, cardiac index, HDL, and FIB. Furthermore, very low-quality evidence was observed for LVEF and LVEDD. The main reasons for downgrading the quality of evidence were the poor methodological quality of the included RCTs, high heterogeneity among the studies, small sample sizes, potential publication bias, and the number of including RCTs.

**TABLE 2:** Summary of findings.

NO.	Study design	Certainty assessment					Summary of results						Importance
		Risk of bias	Inconsistency	Indirectness	Imprecision	Others	No of patients		Effect (95%CI)		Certainty		
							T	C	Relative	Absolute			
Clinical efficacy													
30	RCT	not Serious	not Serious	not Serious	not Serious	Serious ^a^	1,534	1,478	RR = 1.25 (1.20–1.29)	-	⊕⊕⊕○	moderate	Critical
Angina duration													
9	RCT	not Serious	Serious ^b^	not Serious	not Serious	not Serious	349	350	-	MD = −1.58 (−2.18 to −0.97)	⊕⊕⊕○	moderate	Critical
Angina attack frequency													
10	RCT	not Serious	Serious ^b^	not serious	not Serious	Serious ^a^	380	380	-	MD = −2.23 (−3.00 to −1.46)	⊕⊕○○	low	Critical
LVEF													
3	RCT	Serious ^c^	Serious ^b^	not serious	Serious ^d^	Serious ^e^	98	98		MD = 8.17 (5.74–10.6)	⊕○○○	Very low	Important
LVEDD													
3	RCT	Serious ^c^	Serious ^b^	not serious	Serious ^d^	Serious ^e^	98	98	-	MD = −5.12 (−5.95 to −4.28)	⊕○○○	Very low	Important
CI													
2	RCT	not Serious	not Serious	not serious	Serious ^d^	Serious ^e^	68	68	-	MD = 0.31 (0.16–0.45)	⊕⊕○○	Low	Important
BNP													
4	RCT	not Serious	not Serious	not serious	Serious ^d^	Serious ^e^	172	172	-	MD = −21.81 (−27.86 to −15.77)	⊕⊕○○	Low	Important
ECG efficacy													
20	RCT	not Serious	not Serious	not Serious	not serious	Serious ^a^	1,070	1,029	RR = 1.35 (1.27–1.42)	-	⊕⊕⊕○	moderate	Important
CRP													
15	RCT	not Serious	Serious ^b^	not serious	not serious	not serious	729	703	-	MD = −2.06 (−2.58 to −1.54)	⊕⊕⊕○	moderate	Important
TC													
9	RCT	not Serious	Serious ^b^	not serious	not serious	not serious	460	437	-	MD = −1.02 (−1.48 to −0.55)	⊕⊕⊕○	moderate	Important
TG													
8	RCT	not Serious	Serious ^b^	not serious	not serious	not serious	418	399	-	MD = −0.81 (−1.06 to −0.57)	⊕⊕⊕○	moderate	Important
LDL													
6	RCT	not Serious	Serious ^b^	not serious	not serious	not serious	316	297	-	MD = −1.12 (−1.48 to −0.75)	⊕⊕⊕○	moderate	Important
HDL													
4	RCT	not Serious	not Serious	not serious	Serious ^d^	Serious ^e^	143	140	-	MD = 0.62 (0.53–0.71)	⊕⊕○○	Low	Important
FIB													
12	RCT	not Serious	Serious ^b^	not serious	not serious	Serious ^a^	391	351	-	MD = −0.43 (−0.57 to −0.30)	⊕⊕○○	Low	Important
PV													
15	RCT	not Serious	Serious ^b^	not serious	not serious	not serious	929	892	-	MD = −0.37 (−0.49 to −0.25)	⊕⊕⊕○	moderate	Important
HCT													
6	RCT	not Serious	Serious ^b^	not serious	not serious	not serious	305	295	-	MD = −4.72 (−6.78 to −2.66)	⊕⊕⊕○	moderate	Important
HS													
6	RCT	not Serious	Serious ^b^	not serious	not serious	not serious	380	338	-	MD = −0.90 (−1.35 to −0.46)	⊕⊕⊕○	moderate	Important

Abbreviations: RCT, randomized controlled trial; MD, mean difference; RR, relative risk; CI, confidence interval.

^a^Downgrade by one level: There was a risk of publication bias.

^b^ Downgrade by one level: Heterogeneity among the studies was fairly high.

^c^Downgrade by one level: More than 25% of the studies were those with a higher risk of overall bias.

^d^ Downgrade by one level: The optimal information sample size was less than 400 participants.

^e^ Downgrade by one level: The number of RCTs, was s less than 6.

### 3.7 Evaluation of this systematic review

Two evaluators (FCN and ZCY) who had no conflict of interest with this study used AMSTAR-2 and ROBIS to evaluate the methodological quality and risk of bias in this study. The results showed that no significant methodological errors were found in this systematic review, and the risk of bias was low (Supplementary Table S13, S14).

## 4 Discussion

### 4.1 Summary of evidence

This study evaluated the efficacy and safety of SXNI combination therapy in treating CHD. A total of 39 trials involving 3,779 patients with CHD were included. The meta-analysis results indicated that SXNI combination therapy had a significant impact on improving clinical efficacy and decreasing the frequency and duration of angina, suggesting that SXNI significantly improve the clinical symptoms and the quality of life of patients with CHD. Furthermore, the results of the cardiac function indexes proved that SXNI combination therapy significantly decreased LVEDD and increased CI, CO, and LVEF, suggesting that SXNI could effectively improve heart function and avoid the development of chronic heart failure in patients with CHD. Given that blood lipids indexes and thrombosis factors are strongly associated with the severity of CHD and all-cause mortality, the meta analysis results indicated SXNI combination therapy had a significant impact on improving blood lipids indexes (e.g., increased HDL, decreased TC, TGD and LDL) and thrombosis factors (e.g., decreased FIB, PV, HCT, and HS), which indicates that SXNI could reduce the severity of disease and improve the quality of life of patients. At the same time, the therapy also resulted in decreased CRP levels and increased ECG efficacy rate and BNP levels. In terms of safety, based on available evidence, we can only conservatively assume that SXNI does not increase the occurrence of adverse reactions. In summary, SXNI could improve the symptoms, signs and quality of life of patients, reduce the severity of CHD, and improve clinical efficacy. However, the level of evidence was very low to moderate due to the potential publication bias and heterogeneity.

### 4.2 Applicability of evidence

Similar to the results of this study, an animal experiment found that SXNI inhibits oxidative stress and endoplasmic reticulum stress (ERS). This, in turn, regulates the TLR4/NF-κB pathway, leading to a reduction in inflammatory responses and a reduced risk of thrombosis (Wang et al., 2019). A cell experiment found that SXNI can reduce the generation of superoxide and inhibit apoptosis, resulting in a reduction in infarct size and an improvement in cardiac function (Li et al., 2019). In addition, SXNI exhibited significant improvements in ST-segment changes in the ECG of rats with myocardial ischemia and reduced the incidence of arrhythmia (Guo et al., 2012). Another animal experiment discovered that ginkgo flavonol glycosides (GFGs), the active ingredient of SXNI, mitigated heart injury in mice by downregulating the tumor necrosis factor (TNF)-like weak inducer of apoptosis/fibroblast growth factor-inducible 14 (TWEAK/Fn14) axis and reducing the generation of oxygen-free radicals (Xiao et al., 2019). Furthermore, A study based on RNA-seq and network pharmacological analysis showed that SXNI effectively protects heart from injuries via a common Tnfrsf12a-mediated pathway involving atherosclerosis signaling and inflammatory response (Lyu et al., 2018). Overall, SXNI can protect cardiomyocytes and reduce the size of the myocardial infarct by regulating blood lipids, reducing oxidative stress, reducing inflammatory factors, and regulating platelet aggregation, thus improving cardiac function and symptoms of CHD.

### 4.3 Quality of evidence

We assessed the quality of evidence for outcome indicators to guide clinical practice. First, the majority of studies had an acceptable risk of bias, except for one study which was deemed to have an extreme risk of bias. We downgraded the risk level by 1 grade if more than 25% of participants in a comparison were from studies with a high risk of overall bias. Second, heterogeneity was used to evaluate the inconsistency, and significant heterogeneity was found in 12 outcome indicators. Despite the high degree of heterogeneity among the studies, we chose to reduce the level by 1 grade given that the sensitivity analysis confirmed the robustness of the conclusions. Third, no major differences were found in the characteristics (e.g., age, sex, diagnosis, and treatment parameters) of the different studies, and no serious issues of indirectness were found. Fourth, in term of imprecision, we defined the optimal information sample size of the evidence as 400, and we would chose to reduce the level by 1 grade if the total sample size was less than 400 participants. Fifth, the asymmetry of the funnel plot and the results of the Egger’s test indicated potential publication bias in four outcome indicators. Therefore, we reduced the level by 1 grade. Furthermore, if the number of RCTs for an outcome indicator was less than 6, we also reduced the level by 1 grade.

Overall, all outcome indicators had at least one downgrade factor. The quality of evidence for 10 outcome indicators were rated as “moderate”, 5 outcome indicators were rated as “low”, and 2 outcome indicators were rated as “very low”. In addition, two outcome indicators (CO and adverse reactions) were not assessed for the quality of their evidence as only descriptive analyses were performed.

### 4.4 Secondary findings

We assessed the impact of treatment duration, dose of SXNI, mode of administration, and subtypes of disease on the clinical efficacy. Notably, the results from subgroup analyses were not fully consistent. Both SAP and UAP are the most representative disease subtypes of CHD. However, we found SXNI combination therapy showed a positive effect in the treatment of UAP but not on SAP. We surmised that the differences in efficacy across disease subtypes could be attributed to the number of included studies (only 1 study recruited patients with SAP, but 14 studies recruited patients with UAP). Therefore, it is crucial to conduct rigorously designed and higher-quality RCTs to confirm the effectiveness of SXNI in the treatment of SAP. Furthermore, our findings indicated that SXNI could enhance the clinical efficacy regardless of the duration of treatment (less than 2 weeks, 2 weeks, or over 2 weeks), SXNI dose (less than 20 mL/day, 20 mL/day, or over 20 mL/day), and mode of administration (intravenous injection or intramuscular injection).

In view of the high heterogeneity of outcome indicators, sensitivity analysis, subgroup analysis, and meta-regression tests were used to eliminate the heterogeneity. We found that the source of clinical heterogeneity in HDL was due to the inconsistency of treatment duration, and the higher risk of overall bias in the included studies was the source of methodological heterogeneity in LVEDD and LVEF. The meta-regression analysis showed that mean age may be the source of heterogeneity in PV, but the age-based subgroup analysis did not show significantly decreased levels of heterogeneity among the studies. Despite our efforts to eliminate heterogeneity, some outcome indicators still exhibited high levels of heterogeneity, prompting us to interpret the results with cautions for clinical practice when involving in the effect of SXNI for CHD by decreasing the frequency and duration of angina, decreasing CRP, improving blood lipids (decrease TC, TGD and LDL) and thrombosis factors (e.g., decrease FIB, PV, HCT, and HS).

Additionally, we identified publication bias in the FIB index. Notably, while the sensitivity analysis confirmed the robustness of the existing conclusions, the trim-and-fill analysis found that some RCTs showing negative results remained unpublished, and the existing conclusions may be overturned when the negative trials are published. Therefore, this conclusion needs to be treated with caution.

### 4.5 Advantages and limitations

Previous SRs also showed that SXNI is effective and safe in treating CHD. However, this study has the following advantages over previous studies. First, our study comprehensively evaluated the efficacy of SXNI in CHD by examining 19 outcome indicators. Second, this study was conducted strictly in accordance with PRISMA. Furthermore, ROBIS and AMSTAR-2 were used to evaluate the risk of bias and methodological quality of this SR, which enhanced the credibility of the findings. Third, subgroup analyses were conducted to explore the influence of certain characteristics on the efficacy of treatment, which could have clinically significant. Fourth, we used trim-and-fill analysis to assess the impact of publication bias on the interpretation of the results. Finally, this study avoided the flaws of previous studies and updated the evidence.

However, this study unavoidably has the following limitations. First, the majority of the included studies had a risk of bias which reduced the credibility of the evidence. Second, considering that traditional Chinese medicine is also popular in other countries (Wang et al., 2023), while the studies included in this SR were all conducted in China which may lead to bias. Third, although sensitivity analyses confirmed the robustness of these conclusions, existing conclusions need to be treated with caution due to heterogeneity and publication bias. These issues were not fully addressed and could affect the reliability of the results. Fourth, although long-term efficacy is an important part of clinical evaluation, our study did not evaluate the long-term efficacy of SXNI. Notably, previous studies have shown that administration of Shuxuening in combination with potassium aspartate and magnesium, atorvastatin calcium, Shengmai injection, pantoprazole sodium, or high-dose medication was a risk factor for suspected allergic reactions. Furthermore, the incidence of allergic reaction was also influenced by the vehicle, duration of treatment, single dose, and indicated vs. off-label use (Wang et al., 2018). However, due to the different criteria used to evaluate adverse events in each study, reliable safety conclusions could not be reached.

### 4.6 Implications for practice

Based on the conclusions and limitations of this SR, several beneficial and feasible suggestions can be proposed for future research.

Given the inconsistent findings for different subtypes of CHD, future studies should pay more attention to evaluating the efficacy of SXNI, specifically in subtypes such as SAP. Considering the high mortality rate of CHD, future trials should take long-term efficacy as a part of the outcome indicators. In addition, since this study did not draw an accurate safety conclusion, future studies should standardize the monitoring and recording of adverse reactions. Notably, the trim-and-fill analysis found that several RCTs showing negative findings remained unpublished, which could influence the existing findings. Therefore, the bias of selective reporting should be avoided in the future research. Finally, the effects of treatment duration, mode of administration, and dose of SXNI need to be further investigated.

In terms of clinical study design, more large-sample, multi-center, long-period RCTs should be conducted, and strictly follow the Consolidated Standards of Reporting Trials (CONSORT) guidelines. In addition, the sample size should be reasonably estimated, random allocation and allocation concealment methods should be selected, and blind method should be implemented as far as possible. At the same time, the selection of outcome indicators should be able to fully evaluate the efficacy and safety of SXNI, and the measurement of outcome indicators should use a unified standard.

## 5 Conclusion

This study confirmed that SXNI combination therapy can improve the curative effect, symptoms, signs and pathological indexes of patients with CHD in an all-round and multi-channel manner. However, due to the low quality evidence of the included studies, the overall level of evidence is not high. More studies with high-quality, multi-center, and large-sample sizes are needed to confirm the findings.

## Abbreviations

AMSTAR-2, modified quality assessment scale for systematic reviews; AP, angina pectoris; BNP, brain natriuretic peptide; CHD, coronary heart disease; CI, cardiac index; CO, cardiac output; CRP, C-reactive protein; ECG, electrocardiogram; GRADE, grading of recommendation, assessment, development, and evaluation; FIB, fibrinogen; LVEDD, left ventricular end-diastolic dimension; LVEF, left ventricular ejection fraction; HCT, hematocrit; HDL, high-density lipoprotein; HS, whole blood high shear rate; LDL, low-density lipoprotein; MD, mean difference; PRISMA, referred reporting program for systematic review and meta-analysis; PV, plasma viscosity; RCTs, randomized controlled trials; ROBIS, Risk of bias in systematic reviews tool; RR, relative risk; SAP, stable angina pectoris; SR, systematic review; SXNI, Shuxuening injection; TC, total cholesterol; TG, triglyceride; UAP, unstable angina pectoris; 95%CI, 95% confidence interval.
