# Supplementary material for: Effectiveness of Shuxuening injection in coronary heart disease: a systematic review and meta-analysis
Source: Front Pharmacol. 2023 Sep 18;14:1265603. doi: 10.3389/fphar.2023.1265603 (PMC10544985; doi:10.3389/fphar.2023.1265603)

# Effectiveness of Shuxuening Injection in coronary heart disease: A systematic review and meta-analysis

**Menglong Shi^a,b,1^, Tianye Sun^c,1^, Zhaochen Ji^a,b^, Yucong Ma^a,b^, Min Zhao^d^, Fengwen Yang^a,b**^, Junhua Zhang^a,b*^**

a State Key Laboratory of Component-Based Chinese Medicine, Tianjin University of Traditional Chinese Medicine, Tianjin 301617, China

b Evidence-Based Medicine Center, Tianjin University of Traditional Chinese Medicine, Tianjin 301617, China

c Dongfang Hospital, Beijing University of Chinese Medicine, Beijing 102488, China

d The First Affiliated Hospital of Henan University of CM, Zhengzhou 450000, China

Keywords:

Shuxuening injection

Coronary heart disease

Meta-analysis

Systematic review

Randomized controlled trial

Abbreviations: AMSTAR-2, Modified Quality Assessment Scale for Systematic Reviews; AP, angina pectoris; BNP, brain natriuretic peptide; CHD, Coronary heart disease; CI, cardiac index; CO, cardiac output; CRP, C-reactive protein; ECG, electrocardiogram; GRADE, Grading of Recommendation, Assessment, Development, and Evaluation; FIB, fibrinogen; LVEDD, left ventricular end-diastolic dimension; LVEF, left ventricular ejection fraction; HCT, hematocrit; HDL, and high-density lipoprotein; HS, whole blood high shear rate; LDL, low-density lipoprotein; MD, mean difference; PRISMA, referred Reporting Program for Systematic Review and Meta-Analysis; PV, plasma viscosity; RCTs, [randomized controlled trials](https://www.sciencedirect.com/topics/medicine-and-dentistry/randomized-controlled-trial); ROBIS, Risk of bias in systematic reviews tool; RR, relative risk; SAP, [stable angina pectoris](https://www.sciencedirect.com/topics/medicine-and-dentistry/stable-angina-pectoris); SR, systematic review; SXNI, Shuxuening injection; TC, total cholesterol; TG, triglyceride; UAP, [unstable angina pectoris](https://www.sciencedirect.com/topics/medicine-and-dentistry/unstable-angina-pectoris); 95%CI, 95% confidence interval

* Corresponding author at: State Key Laboratory of Component-Based Chinese Medicine, Tianjin University of Traditional Chinese Medicine, Tianjin 301617, China

** Co-corresponding author at: State Key Laboratory of Component-Based Chinese Medicine, Tianjin University of Traditional Chinese Medicine, Tianjin 301617, China

E-mail addresses: zjhtcm@foxmail.com (J. Zhang), 13682027022@163.com (F. Yang).

^1^These authors contributed equally.

**Supplementary Materials**

**catalogue**

[Effectiveness of Shuxuening Injection in coronary heart disease: A systematic review and meta-analysis 1](#_Toc13157)

[Supplementary Materials 2](#_Toc25992)

[catalogue 2](#_Toc780)

[Table S1 PRISMA checklist of the meta-analysis. 1](#_Toc10729)

[Table S2 Botanical drugs included and traditional effects of Shuxuening injection 6](#_Toc9605)

[Table S3 Search strategies. 7](#_Toc22209)

[Table S4 The criteria of Outcome indicators. 10](#_Toc14542)

[Table S5 The reasons of excluded 12 studies. 11](#_Toc19349)

[Table S6 Trim-and-fill test of clinical efficacy rate. 12](#_Toc23015)

[Table S7 Trim-and-fill test of the EGG efficacy. 13](#_Toc18093)

[Table S8 Meta-regression of the CRP. 14](#_Toc13850)

[Table S9 Meta-regression of the blood lipid. 15](#_Toc4034)

[Table S10 Trim-and-fill test of the FIB. 16](#_Toc28529)

[Table S11 Meta-regression of the thrombosis factors. 17](#_Toc7460)

[Table S2 Details of the adverse event occurred in the included studies. 18](#_Toc13751)

[Table S13 Methodological quality assessment 19](#_Toc12073)

[Table S13 the risk of bias in this systematic review. 21](#_Toc16579)

[Fig. S1 Trim-and-fill test analysis to assess the effect of publication bias on the interpretation of the results. 24](#_Toc31704)

[Fig. S2 Effect of SXN on heart function. 24](#_Toc32221)

[Fig. S3 Effect of SXN on ECG efficacy. 25](#_Toc5426)

[Fig. S4 Trim-and-fill test analysis to assess the effect of publication bias on the interpretation of the results. 25](#_Toc4471)

[Fig. S5 Effect of SXN on CRP. 26](#_Toc18058)

[Fig. S6 Effect of SXN on BNP. 26](#_Toc6041)

[Fig. S7 Effect of SXN on TC. 27](#_Toc18885)

[Fig. S8 Effect of SXN on TG. 27](#_Toc8359)

[Fig.S9 Effect of SXN on LDL. 27](#_Toc31614)

[Fig. S10 Effect of SXN on HDL. 28](#_Toc5490)

[Fig. S11 Effect of SXN on FIB. 28](#_Toc11613)

[Fig. S12 Effect of SXN on PV. 29](#_Toc17584)

[Fig. S13 Subgroup analysis of the PV based on the mean age. 29](#_Toc28716)

[Fig. S14 Effect of SXN on HCT. 30](#_Toc11597)

[Fig. S15 Effect of SXN on HS. 30](#_Toc15199)

**Table S1 PRISMA checklist of the meta-analysis.**

| **Section and Topic** | **Item #** | **Checklist item** | **Location where item is reported** |
| --- | --- | --- | --- |
| **TITLE** | | |  |
| Title | 1 | Identify the report as a systematic review. | P1 |
| **ABSTRACT** | | |  |
| Abstract | 2 | See the PRISMA 2020 for Abstracts checklist. | P3 |
| **INTRODUCTION** | | |  |
| Rationale | 3 | Describe the rationale for the review in the context of existing knowledge. | P2-P3 |
| Objectives | 4 | Provide an explicit statement of the objective(s) or question(s) the review addresses. | P2-P3 |
| **METHODS** | | |  |
| Eligibility criteria | 5 | Specify the inclusion and exclusion criteria for the review and how studies were grouped for the syntheses. | P4-P5 |
| Information sources | 6 | Specify all databases, registers, websites, organisations, reference lists and other sources searched or consulted to identify studies. Specify the date when each source was last searched or consulted. | P4 |
| Search strategy | 7 | Present the full search strategies for all databases, registers and websites, including any filters and limits used. | P4 and Supplementary TableS2 |
| Selection process | 8 | Specify the methods used to decide whether a study met the inclusion criteria of the review, including how many reviewers screened each record and each report retrieved, whether they worked independently, and if applicable, details of automation tools used in the process. | P5 |
| Data collection process | 9 | Specify the methods used to collect data from reports, including how many reviewers collected data from each report, whether they worked independently, any processes for obtaining or confirming data from study investigators, and if applicable, details of automation tools used in the process. | P4-P5 |
| Data items | 10a | List and define all outcomes for which data were sought. Specify whether all results that were compatible with each outcome domain in each study were sought (e.g. for all measures, time points, analyses), and if not, the methods used to decide which results to collect. | P5 |
|  | 10b | List and define all other variables for which data were sought (e.g. participant and intervention characteristics, funding sources). Describe any assumptions made about any missing or unclear information. | P5 |
| Study risk of bias assessment | 11 | Specify the methods used to assess risk of bias in the included studies, including details of the tool(s) used, how many reviewers assessed each study and whether they worked independently, and if applicable, details of automation tools used in the process. | P5 |
| Effect measures | 12 | Specify for each outcome the effect measure(s) (e.g. risk ratio, mean difference) used in the synthesis or presentation of results. | P5-P6 |
| Synthesis methods | 13a | Describe the processes used to decide which studies were eligible for each synthesis (e.g. tabulating the study intervention characteristics and comparing against the planned groups for each synthesis (item #5)). | P5 |
|  | 13b | Describe any methods required to prepare the data for presentation or synthesis, such as handling of missing summary statistics, or data conversions. | P5 |
|  | 13c | Describe any methods used to tabulate or visually display results of individual studies and syntheses. | P5-P6 |
|  | 13d | Describe any methods used to synthesize results and provide a rationale for the choice(s). If meta-analysis was performed, describe the model(s), method(s) to identify the presence and extent of statistical heterogeneity, and software package(s) used. | P5-P6 |
|  | 13e | Describe any methods used to explore possible causes of heterogeneity among study results (e.g. subgroup analysis, meta-regression). | P5 |
|  | 13f | Describe any sensitivity analyses conducted to assess robustness of the synthesized results. | P5 |
| Reporting bias assessment | 14 | Describe any methods used to assess risk of bias due to missing results in a synthesis (arising from reporting biases). | P6 |
| Certainty assessment | 15 | Describe any methods used to assess certainty (or confidence) in the body of evidence for an outcome. | P6 |
| **RESULTS** | | |  |
| Study selection | 16a | Describe the results of the search and selection process, from the number of records identified in the search to the number of studies included in the review, ideally using a flow diagram. | P6 and Figure 1 |
|  | 16b | Cite studies that might appear to meet the inclusion criteria, but which were excluded, and explain why they were excluded. | P6 and Table S4 |
| Study characteristics | 17 | Cite each included study and present its characteristics. | P7 and Table1 |
| Risk of bias in studies | 18 | Present assessments of risk of bias for each included study. | P7 and Figure 2 |
| Results of individual studies | 19 | For all outcomes, present, for each study: (a) summary statistics for each group (where appropriate) and (b) an effect estimate and its precision (e.g. confidence/credible interval), ideally using structured tables or plots. | P7-P12 |
| Results of syntheses | 20a | For each synthesis, briefly summarise the characteristics and risk of bias among contributing studies. | P7-P12 |
|  | 20b | Present results of all statistical syntheses conducted. If meta-analysis was done, present for each the summary estimate and its precision (e.g. confidence/credible interval) and measures of statistical heterogeneity. If comparing groups, describe the direction of the effect. | P7-P12 |
|  | 20c | Present results of all investigations of possible causes of heterogeneity among study results. | P7-P12 |
|  | 20d | Present results of all sensitivity analyses conducted to assess the robustness of the synthesized results. | P7-P12 |
| Reporting biases | 21 | Present assessments of risk of bias due to missing results (arising from reporting biases) for each synthesis assessed. | P7-P12 |
| Certainty of evidence | 22 | Present assessments of certainty (or confidence) in the body of evidence for each outcome assessed. | P13 and Table2 |
| **DISCUSSION** | | |  |
| Discussion | 23a | Provide a general interpretation of the results in the context of other evidence. | P13-P14 |
|  | 23b | Discuss any limitations of the evidence included in the review. | P13-P14 |
|  | 23c | Discuss any limitations of the review processes used. | P15-P16 |
|  | 23d | Discuss implications of the results for practice, policy, and future research. | P15-P16 |
| **OTHER INFORMATION** | | |  |
| Registration and protocol | 24a | Provide registration information for the review, including register name and registration number, or state that the review was not registered. | P4 |
|  | 24b | Indicate where the review protocol can be accessed, or state that a protocol was not prepared. | P4 |
|  | 24c | Describe and explain any amendments to information provided at registration or in the protocol. | P4 |
| Support | 25 | Describe sources of financial or non-financial support for the review, and the role of the funders or sponsors in the review. | P16 |
| Competing interests | 26 | Declare any competing interests of review authors. | P16 |
| Availability of data, code and other materials | 27 | Report which of the following are publicly available and where they can be found: template data collection forms; data extracted from included studies; data used for all analyses; analytic code; any other materials used in the review. | Supplementary |

**Table S2 Botanical drugs included and traditional effects of Shuxuening injection**

| Name | Source species | Main component | Properties | Merdians | Traditional efficacy |
| --- | --- | --- | --- | --- | --- |
| Shuxuening injection | Ginkgo biloba | ginkgo flavonoids, ginkgolid | neutral,Sweet, Bitter | Heart, Lung | invigorating the blood circulation |

**Table S3 Search strategies.**

| **#** | **Searches** |
| --- | --- |
| **Search strategies of CNKI (Searched from inception to Jun. 6, 2023 and found 394 literature)** | |
| **#1** | TKA=('动脉粥样硬化性心脏病'+'动脉粥样硬化性心血管疾病'+'冠心病'+'动脉疾病'+'冠状动脉'+'动脉硬化'+'心绞痛'+'心肌梗死'+'心梗'+'心肌缺血')*('舒血宁'+'舒血宁注射液')*('随机对照试验'+'临床对照试验'+'随机'+'安慰剂'+'对照'+'盲法'+'试验'+'分组') |
| **Search strategies of Wanfang (Searched from inception to Jun. 6, 2023 and found 398 literature)** | |
| **#1** | 主题: (“动脉粥样硬化性心脏病” or “动脉粥样硬化性心血管疾病” or “冠心病” or “动脉疾病” or “冠状动脉” or “动脉硬化” or “心绞痛” or “心肌梗死” or “心梗” or “心肌缺血”) and (“舒血宁” or “舒血宁注射液”) and (“随机对照试验” or “临床对照试验” or “随机” or “安慰剂” or “对照” or “盲法” or “试验” or “分组”) |
| **Search strategies of VIP (Searched from inception to Jun. 6, 2023 and found 429 literature)** | |
| **#1** | M=(动脉粥样硬化性心脏病 OR 动脉粥样硬化性心血管疾病 OR 冠心病 OR 动脉疾病 OR 动脉硬化 OR 心绞痛 OR 心梗 OR 心肌梗死 OR 心肌缺血) AND M=(舒血宁 OR 舒血宁注射液) AND R=(随机对照试验 OR 临床对照试验 OR 随机 OR 安慰剂 OR 盲法 OR 对照 OR 试验 OR 分组) |
| **Search strategies of SinoMed (Searched from inception to Jun. 6, 2023 and found 494 literature)** | |
| **#1** | ("动脉粥样硬化性心脏病" OR "动脉粥样硬化性心血管疾病" OR "冠心病" OR "动脉疾病" OR "冠状动脉" OR "动脉硬化" OR "心绞痛" OR "心梗" OR "心肌梗死" OR "心肌缺血") AND ("舒血宁" OR "舒血宁注射液") AND ("随机对照试验" OR "临床对照试验" OR "随机" OR "安慰剂" OR "盲法" OR "对照" OR "试验" OR "分组") |
| **Search strategies of PubMed (Searched from inception to Jun. 6, 2023 and found 2 literature)** | |
| **#1** | ((((((CHD[Title/Abstract]) OR (Coronary Disease*[Title/Abstract])) OR (Disease*, Coronary[Title/Abstract])) OR (Coronary Heart Disease*[Title/Abstract])) OR (Disease*, Coronary Heart[Title/Abstract])) OR (Heart Disease*, coronary[Title/Abstract])) OR ("Coronary Disease"[Mesh]) |
| **#2** | ((((("Acute Coronary Syndrome"[Mesh]) OR (Acute Coronary Syndrome*[Title/Abstract])) OR (Coronary Syndrome*, Acute[Title/Abstract])) OR (Syndrome*, Acute Coronary[Title/Abstract])) OR (ACS[Title/Abstract])) OR (Chronic coronary syndrome*[Title/Abstract]) |
| **#3** | (Chronic coronary syndrome*[Title/Abstract]) OR (CCS[Title/Abstract]) |
| **#4** | ((Angina pectoris[MeSH]) OR (Stenocardia*[Title/Abstract])) OR (Angor Pectoris[Title/Abstract]) |
| **#5** | ((((((((Myocardial Infarction[MeSH]) OR (Infarction*, Myocardial[Title/Abstract])) OR (Myocardial Infarction*[Title/Abstract])) OR (Cardiovascular Stroke*[Title/Abstract])) OR (Stroke*, Cardiovascular[Title/Abstract])) OR (Myocardial Infarct*[Title/Abstract])) OR (Infarct*, Myocardial[Title/Abstract])) OR (Heart Attacks*[Title/Abstract])) OR (MI[Title/Abstract]) |
| **#6** | #1 OR #2 OR #3 OR #4 OR #5 |
| **#7** | (((Shuxuening[Title/Abstract]) OR (Shu xue ning[Title/Abstract])) OR (Shuxue ning[Title/Abstract])) OR (Shu-xue-ning[Title/Abstract]) |
| **#8** | (((((((controlled clinical trial[Publication Type]) OR (Clinical Trial*[Publication Type])) OR (placebo*[Publication Type])) OR (random*[Publication Type])) OR (allocate*[Publication Type])) OR (assign*[Publication Type])) OR (RCT*[Publication Type])) OR ("Randomized Controlled Trial" [Publication Type]) |
| **#9** | #6 AND #7 AND #8 |
| **Search strategies of EMbase (Searched from inception to Jun. 6, 2023 and found 8 literature)** | |
| **#1** | 'coronary heart disease'/exp OR 'CHD':ti,ab OR 'Coronary Disease*':ti,ab OR 'Disease*, Coronary':ti,ab OR 'Coronary Heart Disease*':ti,ab OR 'Disease*, Coronary Heart':ti,ab OR 'Heart Disease*, Coronary':ti,ab |
| **#2** | 'Acute Coronary Syndrome'/exp OR 'Acute Coronary Syndrome*':ti,ab OR 'Coronary Syndrome*, Acute':ti,ab OR 'Syndrome*, Acute Coronary':ti,ab OR 'ACS':ti,ab |
| **#3** | 'Chronic coronary syndrome*':ti,ab OR 'CCS':ti,ab |
| **#4** | 'Angina pectoris'/exp OR 'Stenocardia*':ti,ab OR 'Angor Pectoris':ti,ab |
| **#5** | 'Myocardial Infarction'/exp OR 'Infarction*, Myocardial':ti,ab OR 'Myocardial Infarction*':ti,ab OR 'Cardiovascular Stroke*':ti,ab OR 'Stroke*, Cardiovascular':ti,ab OR 'Myocardial Infarct*':ti,ab OR 'Infarct*, Myocardial':ti,ab OR 'Heart Attack*':ti,ab OR 'MI':ti,ab |
| **#6** | #1 OR #2 OR #3 OR #4 OR #5 |
| **#7** | 'Shuxuening':ti,ab OR 'Shu xue ning':ti,ab OR 'Shuxue ning':ti,ab OR 'Shu-xue-ning':ti,ab |
| **#8** | 'randomized controlled trial':ti,ab OR 'controlled clinical trial':ti,ab OR 'randomized':ti,ab OR 'placebo':ti,ab OR 'random*':ti,ab OR 'allocate*':ti,ab OR 'assign*':ti,ab OR 'RCT*':ti,ab |
| **#9** | #6 AND #7 AND #8 |
| **Search strategies of Web of Science (Searched from inception to Jun. 6, 2023 and found 5 literature)** | |
| **#1** | TS=(coronary heart disease OR (Coronary Disease*) OR (Disease*, Coronary) OR (Coronary Heart Disease*) OR (Disease*, Coronary Heart) OR (angina) OR (Heart Disease*, Coronary)) |
| **#2** | TS=(Acute Coronary Syndrome* OR (Coronary Syndrome*, Acute) OR (Syndrome*, Acute Coronary) OR (ACS)) |
| **#3** | TS=(Chronic coronary syndrome* OR (CCS)) |
| **#4** | TS=(Angina pectoris OR (Stenocardia*) OR (Angor Pectoris)) |
| **#5** | TS=(Myocardial Infarction OR (Infarction*, Myocardial) OR (Myocardial Infarction*) OR (Cardiovascular Stroke*) OR (Stroke*, Cardiovascular) OR (Heart Attack*') OR (MI) OR (Myocardial Infarct*) OR (Infarct*, Myocardial)) |
| **#6** | #1 OR #2 OR #3 OR #4 OR #5 |
| **#7** | TS=(Shuxuening OR (Shu xue ning*, Acute) OR (Shuxue ning) OR (Shu-xue-ning)) |
| **#8** | TS=(randomized controlled trial OR (controlled clinical trial) OR (randomized) OR (placebo) OR (random*) OR (allocate*) OR (MI) OR (assign*) OR (RCT*)) |
| **#9** | #6 AND #7 AND #8 |
| **Search strategies of Cochrane Central Register of Controlled Trials (CENTRAL) (Searched from inception to Jun. 6, 2023 and found 5 literature)** | |
| **#1** | MeSH descriptor: [Coronary Disease] explode all trees  (Coronary Disease*):ti,ab,kw OR (CHD):ti,ab,kw OR (Disease*, Coronary):ti,ab,kw OR (Coronary Heart Disease*):ti,ab,kw OR (Disease*, Coronary Heart):ti,ab,kw OR (Heart Disease*, coronary):ti,ab,kw OR (Coronary Disease):ti,ab,kw |
| **#2** | MeSH descriptor: [Acute Coronary Syndrome] explode all trees  (Acute Coronary Syndrome*):ti,ab,kw OR (Coronary Syndrome*, Acute):ti,ab,kw OR (Syndrome*, Acute Coronary):ti,ab,kw OR (ACS):ti,ab,kw OR (Chronic coronary syndrome*):ti,ab,kw |
| **#3** | (Chronic coronary syndrome*):ti,ab,kw OR (CCS):ti,ab,kw |
| **#4** | MeSH descriptor: [Angina Pectoris] explode all trees  (Stenocardia*):ti,ab,kw OR (Angor Pectoris):ti,ab,kw |
| **#5** | MeSH descriptor: [Myocardial Infarction] explode all trees  (Infarction*, Myocardial):ti,ab,kw OR (Myocardial Infarction*):ti,ab,kw OR (Cardiovascular Stroke*):ti,ab,kw OR (Stroke*, Cardiovascular):ti,ab,kw OR (Myocardial Infarct*):ti,ab,kw OR (Infarct*, Myocardial):ti,ab,kw OR (CHeart Attacks*):ti,ab,kw |
| **#6** | #1 OR #2 OR #3 OR #4 OR #5 |
| **#7** | (Shuxuening):ti,ab,kw OR (Shu xue ning):ti,ab,kw OR (Shuxue ning):ti,ab,kw OR (Shu-xue-ning):ti,ab,kw |
| **#8** | #6 AND #7 |

**Table S4 The criteria of Outcome indicators.**

| **Outcome indicators** | **Clinical efficacy evaluation criteria** |
| --- | --- |
| Clinical effect | Obvious effect: clinical symptoms disappeared, and the ST segment of electrocardiogram basically recovered; Effective: Clinical symptoms basically disappeared, ECG ST segment improved; Ineffective: The symptoms of unstable angina pectoris in these patients were not improved, or even severe (Obvious effect plus effective is defined as total effective). |
| Electrocardiogram (ECG) efficacy | After treatment, the patient's ECG returned to normal or roughly normal. Effective: After treatment, the ST segment of the ECG recovered not less than 0.5 mV, and the T-wave of the main lead became shallow to more than 50% or flat to upright, but it still did not reach the normal level. Ineffective: After treatment, there was no significant change in the patient's ECG before and after treatment |

**Table S5 The reasons of excluded 12 studies.**

| **Excluded studies** | **Reasons** |
| --- | --- |
| (Chu, 2017) | Not the study design |
| (Hou, 2010) | Not the study design |
| (Wu et al., 2006) | Not the target intervention |
| (Fan et al., 2015) | Not the target intervention |
| (Li et al., 2010) | Not the target intervention |
| (Liu, 2011) | Not the target intervention |
| (Si, 2013) | Not the target intervention |
| (Liu et al., 2009) | Not the target intervention |
| (Li, 2013) | Not the target intervention |
| (You, 2021) | Not the target intervention |
| (Deng, 2011) | Not the target patients |
| (Wang and Zhang, 2013) | Not the target patients |

**References:**

Chu, J., 2017, Clinical value of Shuxuening combined with trimetazidine in the treatment of angina pectoris of coronary heart disease. Strait Pharm. J. 29, 100-101.

Deng, Y., 2011, Clinical observation of 80 patients with chronic pulmonary heart disease treated by nitroglycerin combined with Shuxuening injection. MED. J. Chin. People's Health 23, 1866-1867.

Fan, G., Wang, Y., Fu, Q., 2015, Shuxuening Combined with Western Treatment of Angina Pectoris Parallel Randomized Controlled Study. J. Prac. Tradit. Chin. Inter. Med. 29, 113-114.

Hou, J., 2010, Clinical observation of Shuxuening in the treatment of unstable angina pectoris. Guide Chin. Med. 8, 267-268.

Li, C., Chen, Z., You, W., 2010, Curative effect of Shuxuening injection combined with atorvastatin on unstable angina pectoris. Mod. J. Integr. Tradi. Chin. and West. Med. 19, 27-28.

Li, Y., 2013, Effect evaluation of the trimetazidine combined with Shuxuening injection in the treatment of unstable angina. Chin. J. Clinical Rational Drug Use 6, 4-5.

Liu, D., 2011, Clinical Observation of Shuxuening Injection and Trimetazidine on Coronary Heart Disease and Angina Pectoris. Chin. J.f Exper. Tradit. Med. Formu., 258-260.

Liu, H., Tian, K., Qiu, Y., Liu, J., 2009, Clinical observation of treating angina pectoris with Shuxuening. Chin. J.f Pract. Inter. Med., 105-106.

Si, Q., 2013, Clinical effect of Shuxuening on 80 cases of unstable angina pectoris. Practical Clinical J. Integr. Tradi. Chin. and West. Med. 13, 50-51.

Wang, L., Zhang, D., 2013, Clinical observation of Shuxuening injection in the treatment of senile coronary heart failure. J. Mathematical Med. 26, 107-108.

Wu, Z., Liu, Y., Zhang, D., Li, G., 2006, Effect of Shuxuening Injection on NO, NOS, SOD and MDA of Angina Pectoris Patients. Chin. J.F inform. ON Tradi. Chin. Med. 13, 22-24.

You, C., 2021, To observe the clinical effect of trimetazidine combined with Shuxuening in the treatment of angina pectoris of coronary heart disease. World Latest Med. Inform. 21, 181-182, 184.

**Table S6 Trim-and-fill test of clinical efficacy rate.**

| **Outcome** | **Effect-size** | **Effect model** | **Before trim-and-fill** | | **After trim-and-fill** | | **Increased research** |
| --- | --- | --- | --- | --- | --- | --- | --- |
|  |  |  | **Pooled estimate** | **95％CI** | **Pooled estimate** | **95％CI** |  |
| SXNI plus CT *vs.* CT | RR | FE | 1.25 | 1.20 to 1.29 | 1.183 | 1.149 to 1.218 | 10 |
|  |  | RE | 1.22 | 1.19 to 1.27 | 1.184 | 1.147 to 1.222 |  |

Because of the potential publication bias of clinical efficacy rate, we conducted trim-and-fill test analysis to assess the effect of publication bias on the interpretation of the results. The result indicated that this publication bias did not affect the estimates, although several RCTs showing negative findings remained unpublished

**Table S7 Trim-and-fill test of the EGG efficacy.**

| **Outcome** | **Effect-size** | **Effect model** | **Before trim-and-fill** | | **After trim-and-fill** | | **Increased research** |
| --- | --- | --- | --- | --- | --- | --- | --- |
|  |  |  | **Pooled estimate** | **95％CI** | **Pooled estimate** | **95％CI** |  |
| SXN plus CT *vs.* CT | RR | FE | 1.35 | 1.27 to 1.42 | 1.251 | 1.195 to 1.310 | 7 |
|  |  | RE | 1.30 | 1.23 to 1.37 | 1.253 | 1.182 to 1.328 |  |

Because of the potential publication bias of EGG efficacy, we conducted trim-and-fill test analysis to assess the effect of publication bias on the interpretation of the results. The result indicated that this publication bias did not affect the estimates, although several RCTs showing negative findings remained unpublished.

**Table S8 Meta-regression of the CRP.**

| **Characteristic** | **Regression coefficient** | **Standard error** | **t** | **P＞\|t\|** | **95% CI** |
| --- | --- | --- | --- | --- | --- |
| Year of publication | –0.394 | 0.175 | –2.25 | 0.051 | –0.780 to 0.009 |
| Sample size | 0.010 | 0.008 | 1.18 | 0.265 | –0.009 to 0.029 |
| Subtypes of disease | 0.079 | 0.382 | 0.21 | 0.84 | –0.762 to 0.920 |
| Session of treatment | –0.099 | 0.414 | –0.24 | 0.816 | –1.01 to 0.0.812 |
| Mean age | 0.105 | 0.072 | 1.45 | 0.177 | –0.056 to 0.266 |

**Table S9 Meta-regression of the blood lipid.**

| **Characteristic** | **Regression coefficient** | **Standard error** | **t** | **P＞\|t\|** | **95% CI** |
| --- | --- | --- | --- | --- | --- |
| **Total cholesterol (TC)** | | | | | |
| Year of publication | –0.001 | 0.027 | –0.05 | 0.965 | –0.09 to 0.086 |
| Sample size | 0.0002 | 0.006 | 0.03 | 0.978 | –0.03 to 0.03 |
| Subtypes of disease | –0.055 | 0.152 | –0.36 | 0.740 | –0.54 to 0.428 |
| Session of treatment | –0.037 | 0.146 | –0.25 | 0.818 | –0.50 to 0.429 |
| Mean age | 0.007 | 0.011 | 0.64 | 0.588 | –0.04 to 0.054 |
| **Triglyceride (TG)** | | | | | |
| Year of publication | 0.004 | 0.027 | 0.15 | 0.895 | –0.110 to 0.118 |
| Sample size | 0.001 | 0.005 | 0.2 | 0.861 | –0.024 to 0.026 |
| Subtypes of disease | 0.062 | 0.150 | 0.41 | 0.720 | –0.585 to 0.709 |
| Session of treatment | 0.040 | 0.100 | 0.40 | 0.729 | –0.389 to 0.469 |
| Mean age | –0.003 | 0.012 | –0.32 | 0.777 | –0.053 to 0.046 |
| **low-density lipoprotein (LDL)** | | | | | |
| Year of publication | –0.005 | 0.011 | –0.48 | 0.663 | –0.039 to 0.029 |
| Sample size | 0.0004 | 0.004 | 0.09 | 0.933 | –0.018 to 0.019 |
| Subtypes of disease | 0.023 | 0.110 | 0.21 | 0.847 | –0.326 to 0.377 |
| Session of treatment | 0.015 | 0.124 | 0.12 | 0.914 | –0.519 to 0.549 |
| Mean age | 0.004 | 0.016 | 0.25 | 0.828 | –0.063 to 0.701 |

**Table S10 Trim-and-fill test of the FIB.**

| **Outcome** | **Effect-size** | **Effect model** | **Before trim-and-fill** | | **After trim-and-fill** | | **Increased research** |
| --- | --- | --- | --- | --- | --- | --- | --- |
|  |  |  | **Pooled estimate** | **95％CI** | **Pooled estimate** | **95％CI** |  |
| SXN plus CT *vs.* CT | MD | FE | –1.74 | –1.76 to –1.73 | 0.17 | 0.170 to 0.175 | 6 |
|  |  | RE | –1.08 | –1.21 to –0.94 | 0.19 | 0.169 to 0.223 |  |

Because of the potential publication bias of FIB, we conducted trim-and-fill test analysis to assess the effect of publication bias on the interpretation of the results. The result indicated that several RCTs showing negative findings remained unpublished, which could affect the conclusion.

**Table S11 Meta-regression of the thrombosis factors.**

| **Characteristic** | **Regression coefficient** | **Standard error** | **t** | **P＞\|t\|** | **95% CI** |
| --- | --- | --- | --- | --- | --- |
| **Fibrinogen (FIB)** | | | | | |
| Year of publication | 0.025 | 0.742 | 0.33 | 0.747 | –0.141 to 0.190 |
| Sample size | 0.001 | 0.003 | 0.32 | 0.757 | –0.006 to 0.008 |
| Subtypes of disease | 0.986 | 0.235 | 0.42 | 0.684 | –0.426 to 0.623 |
| Session of treatment | –0.419 | 0.244 | –1.72 | 0.117 | –0.963 to 0.125 |
| Mean age | 0.023 | 0.036 | 0.65 | 0.532 | –0.059 to 0.105 |
| **Plasma viscosity (PV)** | | | | | |
| Year of publication | 0.035 | 0.032 | 1.12 | 0.282 | –0.033 to 0.104 |
| Sample size | –0.004 | 0.002 | –2.41 | 0.031 | –0.007 to –0.0004 |
| Subtypes of disease | 0.146 | 0.132 | 1.11 | 0.289 | –0.140 to 0.432 |
| Session of treatment | –0.002 | 0.149 | –0.01 | 0.99 | –0.323 to 0.319 |
| Mean age | –0.022 | 0.010 | –2.28 | 0.049 | –0.045 to –0.0002 |
| **HS** | | | | | |
| Year of publication | –0.038 | 0.106 | –0.35 | 0.741 | –0.332 to 0.256 |
| Sample size | –0.006 | 0.003 | –2.42 | 0.072 | –0.014 to 0.001 |
| Subtypes of disease | –0.344 | 0.435 | –0.79 | 0.474 | –1.553 to 0.866 |
| Session of treatment | –0.072 | 0.289 | –0.25 | 0.815 | –0.873 to 0.729 |
| Mean age | 0.057 | 0.027 | 2.07 | 0.130 | –0.030 to 0.144 |
| **HCT** | | | | | |
| Year of publication | –0.281 | 0.259 | –1.08 | 0.339 | –1.000 to 0.438 |
| Sample size | –0.006 | 0.031 | –0.19 | 0.858 | –0.091 to 0.079 |
| Subtypes of disease | 2.112 | 1.273 | 1.66 | 0.173 | –1.423 to 5.647 |
| Session of treatment | 1.982 | 1.343 | 1.48 | 0.214 | –1.748 to 5.712 |
| Mean age | –0.298 | 0.247 | –1.21 | 0.313 | –1.084 to 0.487 |

**Table S2 Details of the adverse event occurred in the included studies.**

| **Study** | **T** | **C** |
| --- | --- | --- |
| Zhang et al. (2022) | dizziness (1), nausea (1), gastrointestinal reactions (1) | dizziness (2), nausea (1), gastrointestinal reactions (2) |
| Sun et al. (2019) | nausea (2), abnormal liver and kidney function (1) | nausea (3), abnormal liver and kidney function (2) |
| Zhang et al. (2011) | no | no |
| Ma et al. (2012) | nausea (4) | nausea (2), palpitation (1) |
| Wang et al. (2021) | rashes (2), gastrointestinal discomfort (3), dizziness (1) | rashes (1), gastrointestinal discomfort (2), cough (1) |
| Liu et al. (2015) | headache(1), nausea (1), swell (2) | headache(1), nausea (1), swell (1) |
| He et al. (2014) | no | slightly increased of ALT (1) |
| Chai et al. (2010) | rashes (2) | rashes (3) |
| Cai et al. (2021) | rashes (1), gastrointestinal discomfort (3), | rashes (2), gastrointestinal discomfort (1), nausea (2), abnormal liver and kidney function (2) |
| Zhou et al. (2010) | no | no |
| Zhong et al. (2010) | rashes (1) | no |
| Wang et al. (2009) | no | no |
| He et al. (2022) | no | no |
| Yang et al. (2011) | no | no |
| Xu et al. (2012) | no | no |
| Ren et al. (2014) | no | no |
| Sun et al. (2010) | no | facial flushing (2) |
| Zhao et al. (2011) | no | no |
| Gao et al. (2011) | headache(1) | headache(2) |

**Table S13 Methodological quality assessment**

| **Quality evaluation** | **Y/N** | **Location where item is reported** |
| --- | --- | --- |
| 1. Did the research questions and inclusion criteria for the review include the components of PICO? | Y | P4 |
| 1. Did the report of the review contain an explicit statement that the review methods were established prior to the conduct of the review and did the report justify any significant deviations from the protocol? | Y | P4 and without deviations |
| 1. Did the review authors explain their selection of the study designs for inclusion in the review? | Y | P4 |
| 1. Did the review authors use a comprehensive literature search strategy? | Y | Supplement Table S2 |
| 1. Did the review authors perform study selection in duplicate? | Y | P5 |
| 1. Did the review authors perform data extraction in duplicate? | Y | P5 |
| 1. Did the review authors provide a list of excluded studies and justify the exclusions? | Y | P7 and Supplementary Table S4 |
| 1. Did the review authors describe the included studies in adequate detail? | Y | P7-9 |
| 1. Did the review authors use a satisfactory technique for assessing the risk of bias (RoB) in individual studies that were included in the review? | Y | P10 |
| 1. Did the review authors report on the sources of funding for the studies included in the review? | Y | P16 |
| 1. If meta-analysis was performed, did the review authors use appropriate methods for statistical combination of results? | Y | P7-12 |
| 1. If meta-analysis was performed, did the review authors assess the potential impact of RoB in individual studies on the results of the meta-analysis or other evidence synthesis? | Y | P7-12 |
| 1. Did the review authors account for RoB in primary studies when interpreting/discussing the results of the review? | Y | P14-16 |
| 1. Did the review authors provide a satisfactory explanation for, and discussion of, any heterogeneity observed in the results of the review? | Y | P14-16 |
| 1. If they performed quantitative synthesis did the review authors carry out an adequate investigation of publication bias (small study bias) and discuss its likely impact on the results of the review? | Y | P14-16 |
| 1. Did the review authors report any potential sources of conflict of interest, including any funding they received for conducting the review? | N | P16 |

**Table S14 the risk of bias in this systematic review.**

| **Phase 1: Assessing relevance** | | | Y | P1-5 |
| --- | --- | --- | --- | --- |
| **Phase 2. Identifying concerns about bias in the review process** | **Domain 1. Study eligibility criteria** | Q1. Did the review adhere to predefined objectives and eligibility criteria? | Y | P4 |
|  |  | Q2. Were the eligibility criteria appropriate for the review question? | Y | P4 |
|  |  | Q3.Were eligibility criteria unambiguous? | Y | P4 |
|  |  | Q4. Were all restrictions in eligibility criteria based on study characteristics appropriate? | Y | P4 |
|  |  | Q5. Were any restrictions in eligibility criteria based on sources of information appropriate? | Y | P4 |
|  |  | **Risk rating** | Low |  |
|  | **Domain 2. Identification and**  **selection of studies** | Q1. Did the search include an appropriate range of databases/electronic sources for published and unpublished reports? | Y | P4 |
|  |  | Q2. Were methods additional to database searching used to identify relevant reports? | Y | P4 |
|  |  | Q3. Were the terms and structure of the search strategy likely to retrieve as many eligible studies as possible? | Y | P4 and Supplement Table S2 |
|  |  | Q4. Were restrictions based on date.publication format, or language appropriate? | Y | P4 |
|  |  | Q5. Were efforts made to minimize error in selection of studies? | Y | P5 |
|  |  | **Risk rating** | Low |  |
|  | **Domain 3. Data collection and study appraisal** | Q1. Were efforts made to minimize error in data collection? | Y | P5 |
|  |  | Q2. Were sufficient study characteristics available for both review authors and readers to be able to interpret the results? | Y | P5 |
|  |  | Q3. Were all relevant study results collected for use in the synthesis? | Y | P5 |
|  |  | Q4. Was risk of bias (or methodological quality) formally assessed using appropriate criteria? | Y | P5 |
|  |  | Q5. Were efforts made to minimize error in risk of bias assessment? | Y | P5 |
|  |  | **Risk rating** | Low |  |
|  | **Domain 4. Synthesis and findings** | Q1. Did the synthesis include all studies that it should? | Y | P7-12 |
|  |  | Q2. Were all predefined analyses reported or departures explained? | Y | P7-12 |
|  |  | Q3. Was the synthesis appropriate given the nature and similarity in the research questions, study designs, and outcomes across included studies? | Y | P7-12 |
|  |  | Q4. Was between-study variation minimal or addressed in the synthesis? | Y | P7-12 |
|  |  | Q5. Were the findings robust, for example.as demonstrated through funnel plot or sensitivity analyses? | Y | P7-12 |
|  |  | Q6. Were biases in primary studies minimal or addressed in the synthesis? | Y | P7-12 |
|  |  | **Risk rating** | Low |  |
| **Phase 3. Risk of bias in the review** | | Q1. Did the interpretation of findings address all of the concerns identified in domains 1 to 4? | Y | P13-14 |
|  |  | Q2. Was the relevance of identified studies to the review's research question appropriately considered? | Y | P13-14 |
|  |  | Q3. Did the reviewers avoid emphasizing results on the basis of their statistical significance? | Y | P13-14 |
|  | | **Risk rating** | Low |  |

**Fig. S1 Trim-and-fill test analysis to assess the effect of publication bias on the interpretation of the results.**


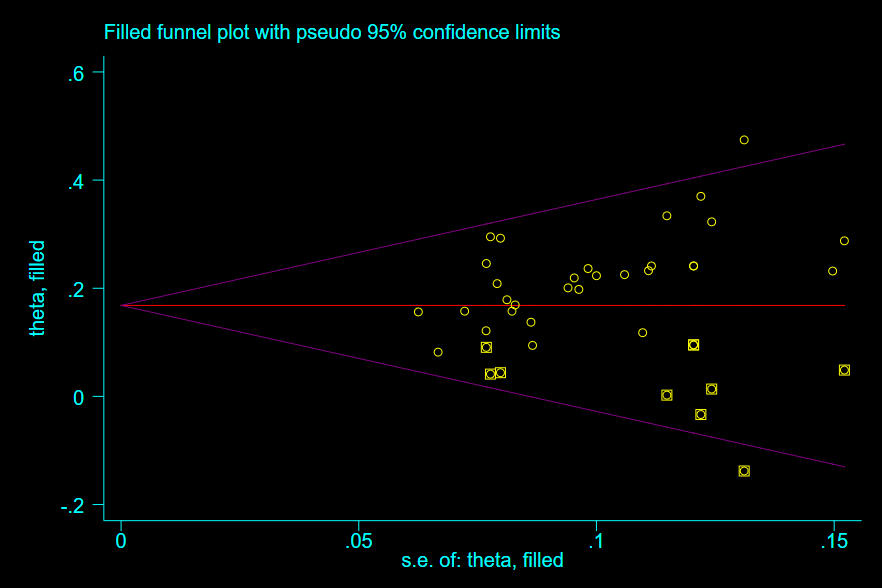


**Fig. S2 Effect of SXN on heart function.**

(A) Forest plot of cardiac index. (B) Forest plot of [LVEF](https://www.sciencedirect.com/topics/medicine-and-dentistry/heart-left-ventricle-ejection-fraction). (C) The result of sensitivity analysis of LVEF. (D) Forest plot of LVEDD. (E)The result of sensitivity analysis of LVEDD.


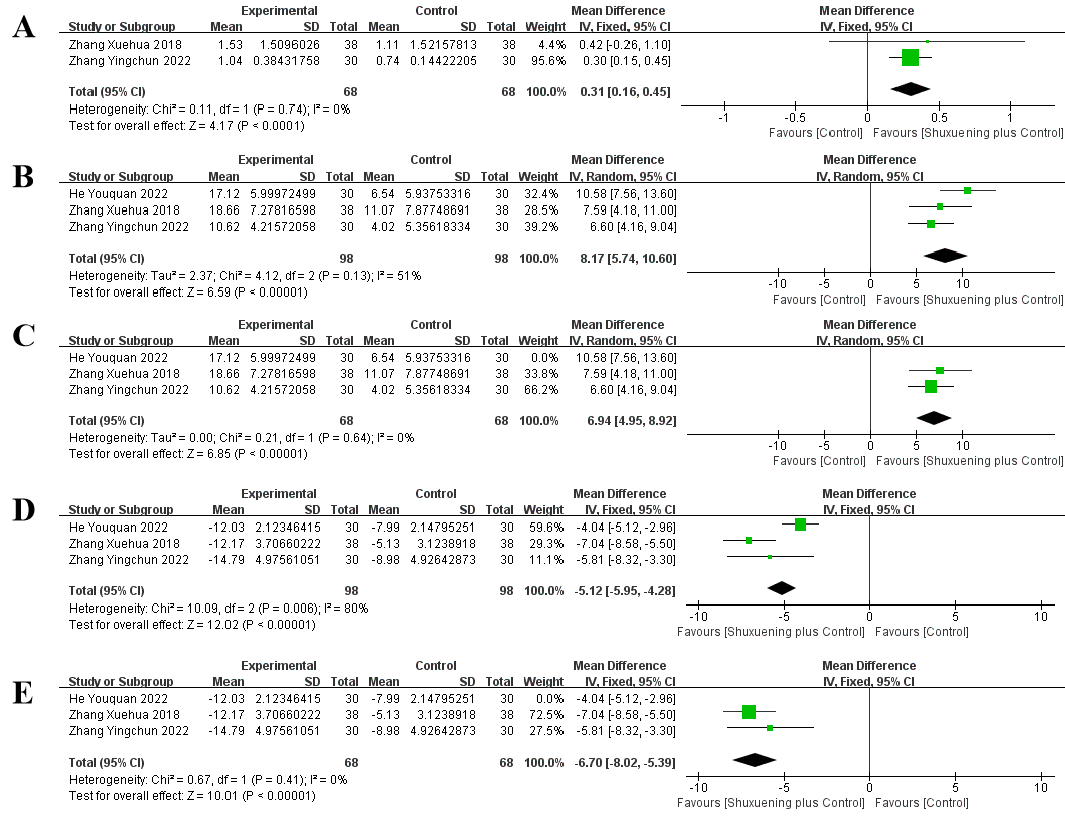


**Fig. S3 Effect of SXN on ECG efficacy.**

(A) Forest plot. (B) Sensitivity analysis revealed the reliability of result. (C) Funnel plots revealed the publication bias. (D) The Egger's test quantified the publication bias.


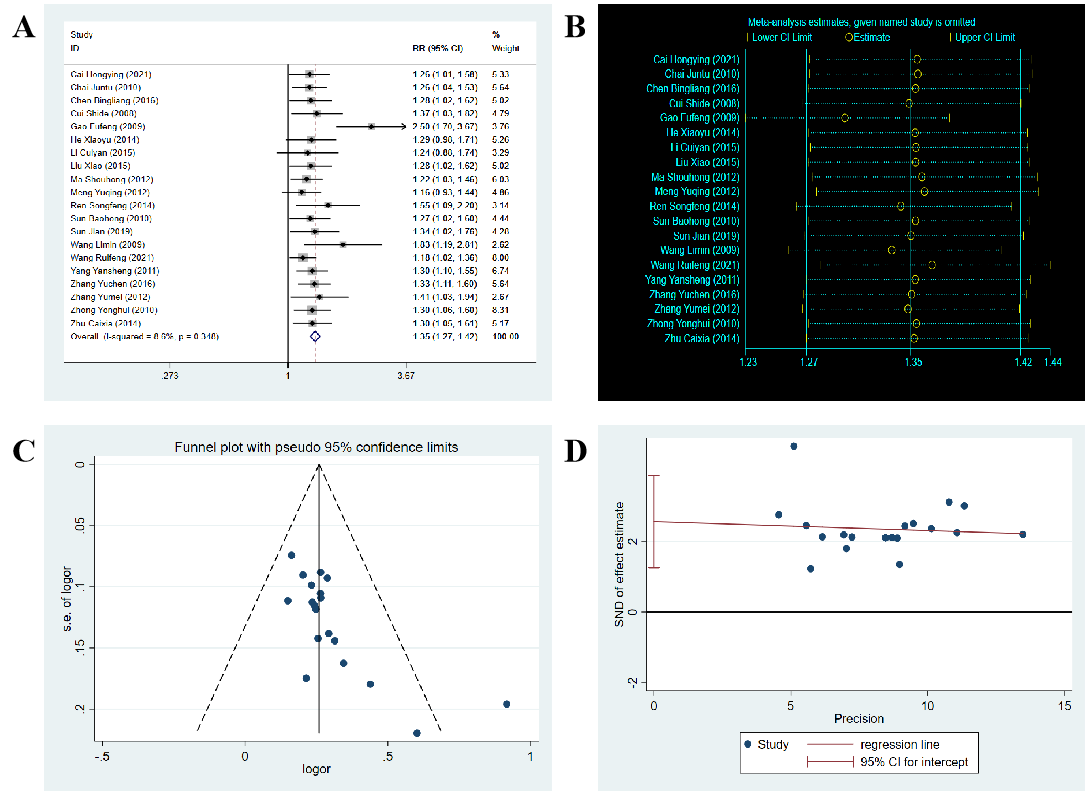


**Fig. S4 Trim-and-fill test analysis to assess the effect of publication bias on the interpretation of the results.**


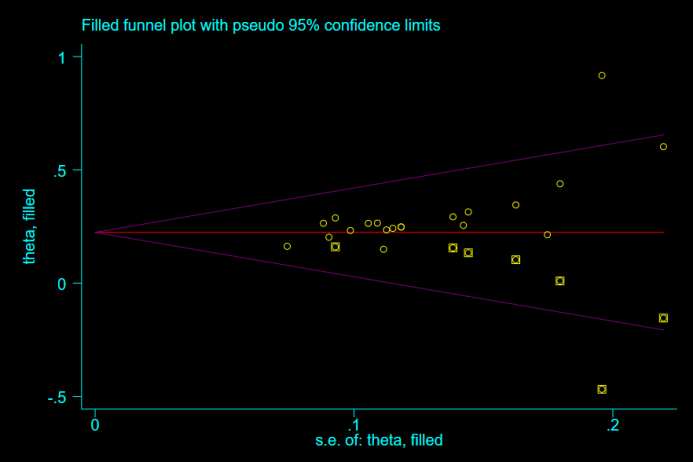


**Fig. S5 Effect of SXN on CRP.**

(A) Forest plot. (B) Sensitivity analysis revealed the reliability of result. (C) Funnel plots revealed the publication bias. (D) The Egger's test quantified the publication bias.


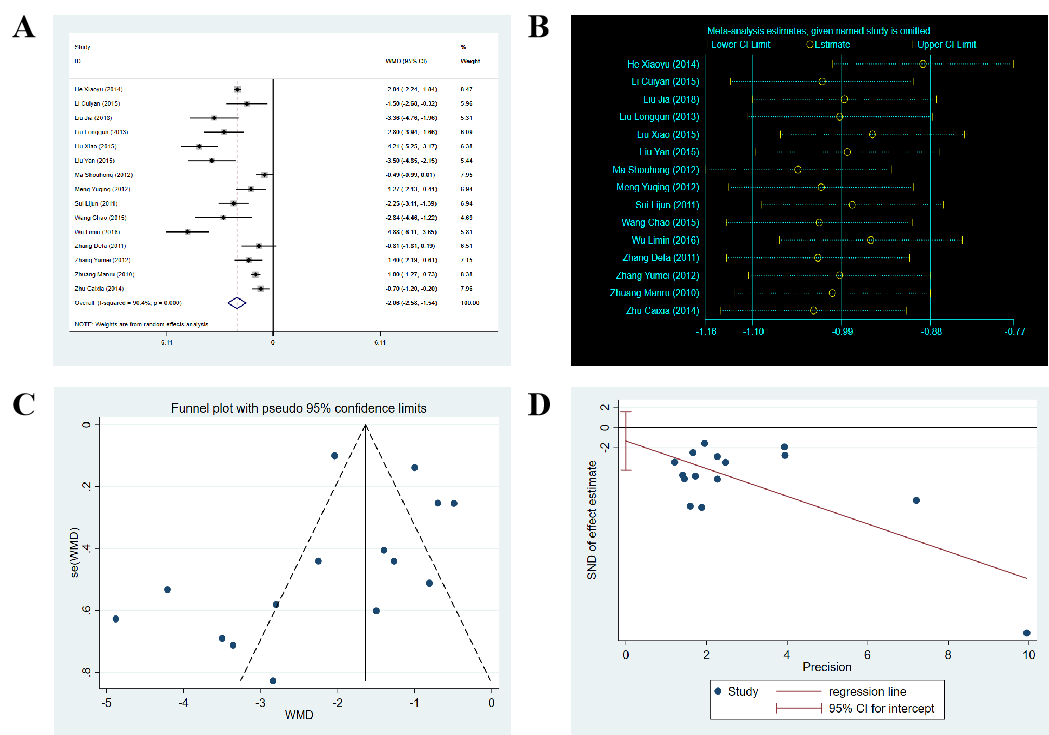


**Fig. S6 Effect of SXN on BNP.**

(A) Forest plot of BNP. (B) Sensitivity analysis revealed the reliability of result.


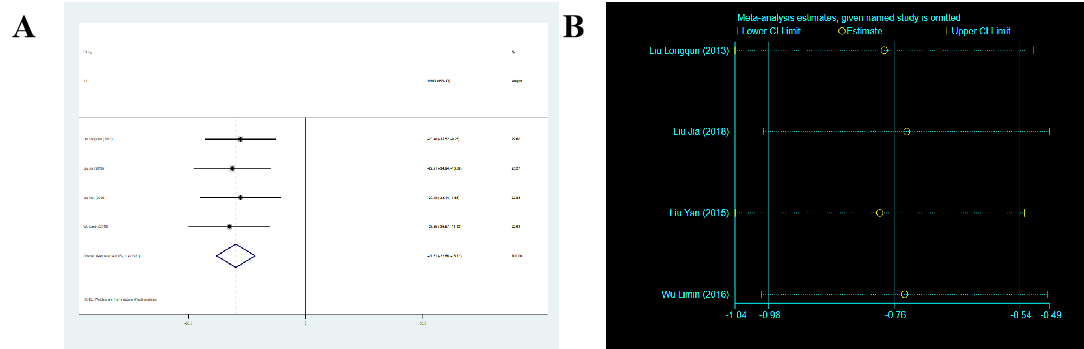


**Fig. S7 Effect of SXN on TC.**

(A) Forest plot of TC. (B) Sensitivity analysis revealed the reliability of result.


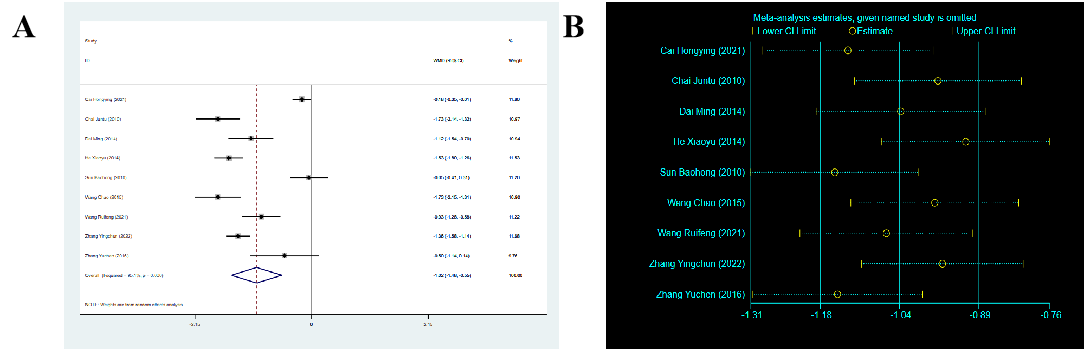


**Fig. S8 Effect of SXN on TG.**

(A) Forest plot of TG. (B) Sensitivity analysis revealed the reliability of result.


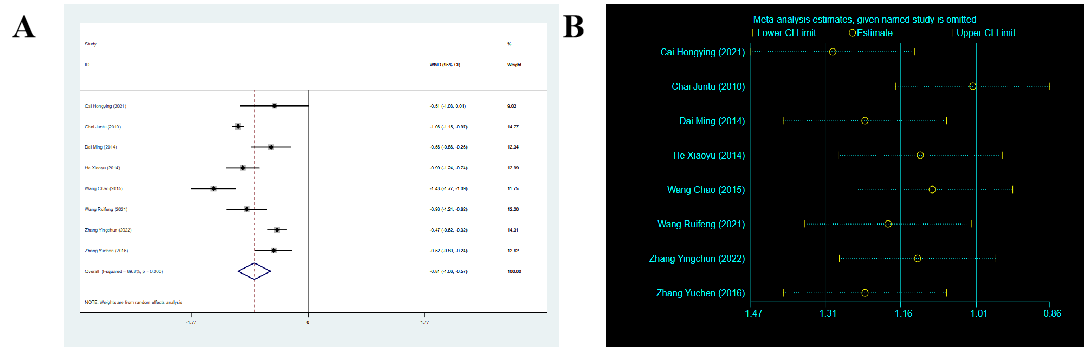


**Fig.S9 Effect of SXN on LDL.**

(A) Forest plot of LDL. (B) Sensitivity analysis revealed the reliability of result.


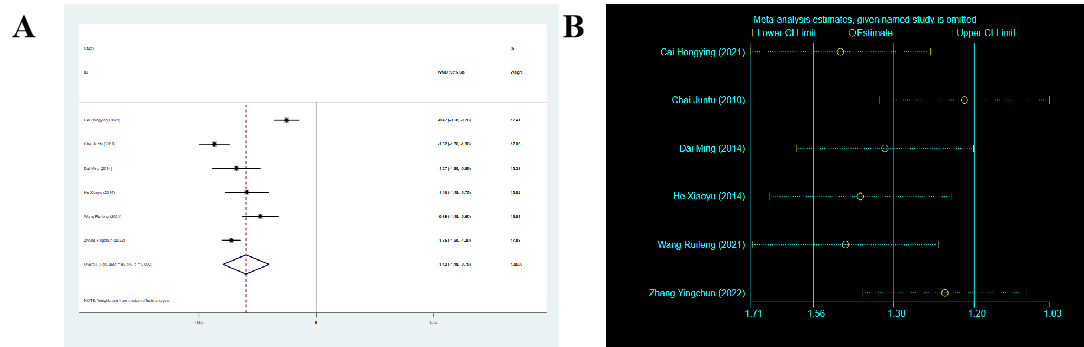


**Fig. S10 Effect of SXN on HDL.**

(A) Forest plot of HDL. (B) Sensitivity analysis revealed the heterogeneity would decline if one RCT was excluded.


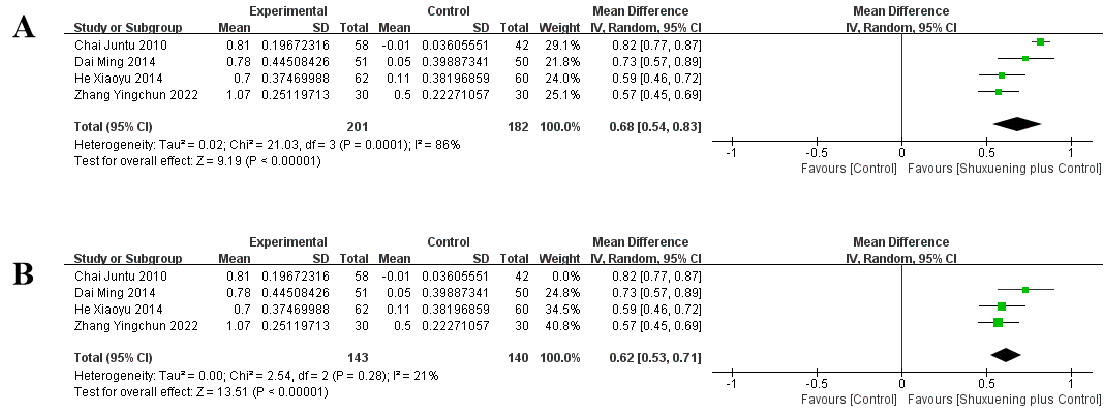


**Fig. S11 Effect of SXN on FIB.**

(A) Forest plot of FIB. (B) Sensitivity analysis revealed the reliability of result. (C) Funnel plots revealed the publication bias. (D) The Egger's test quantified the publication bias.


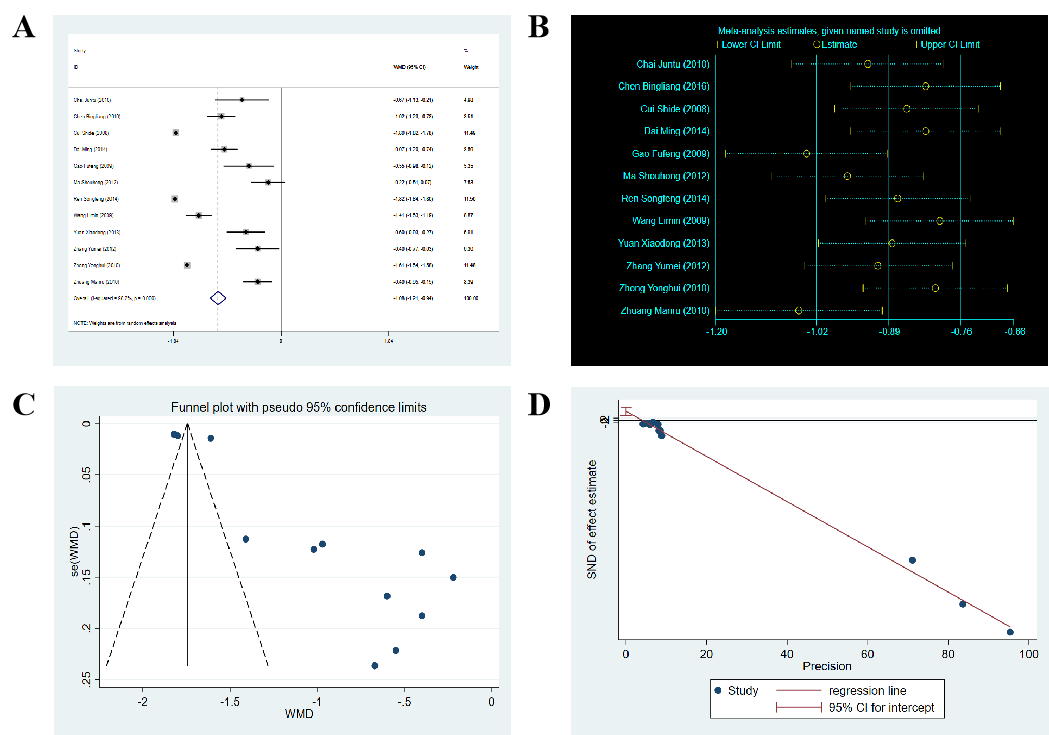


**Fig. S12 Effect of SXN on PV.**

(A) Forest plot of PV. (B) Sensitivity analysis revealed the reliability of result. (C) Funnel plots revealed the publication bias. (D) The Egger's test quantified the publication bias.


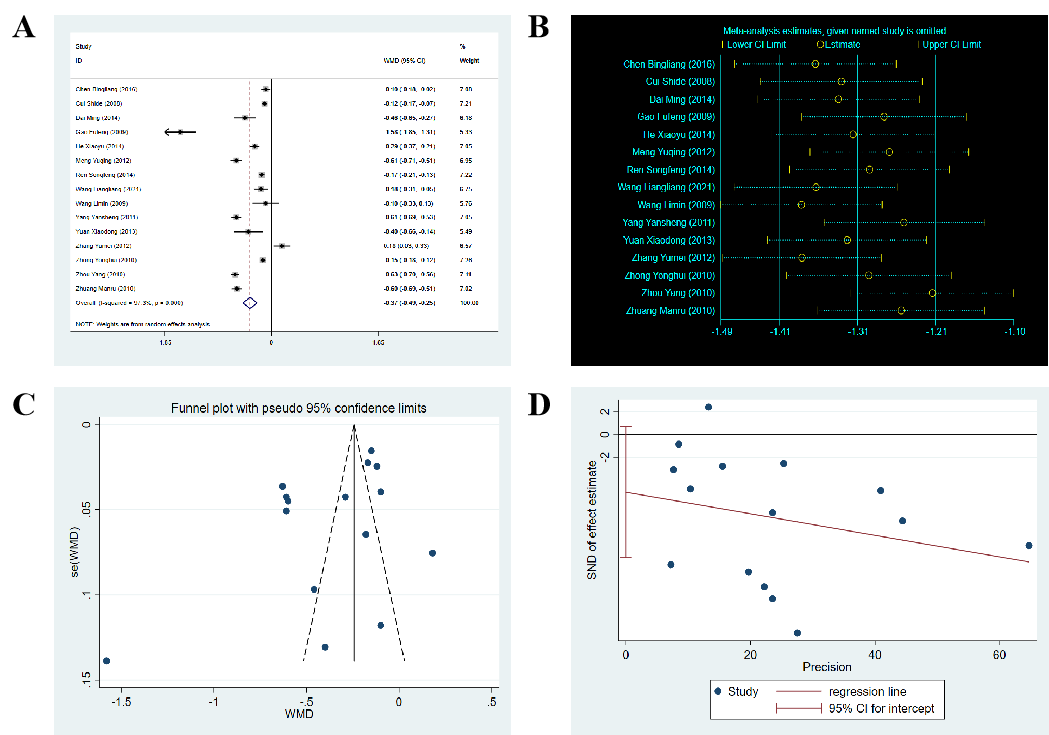


**Fig. S13 Subgroup analysis of the PV based on the mean age.**


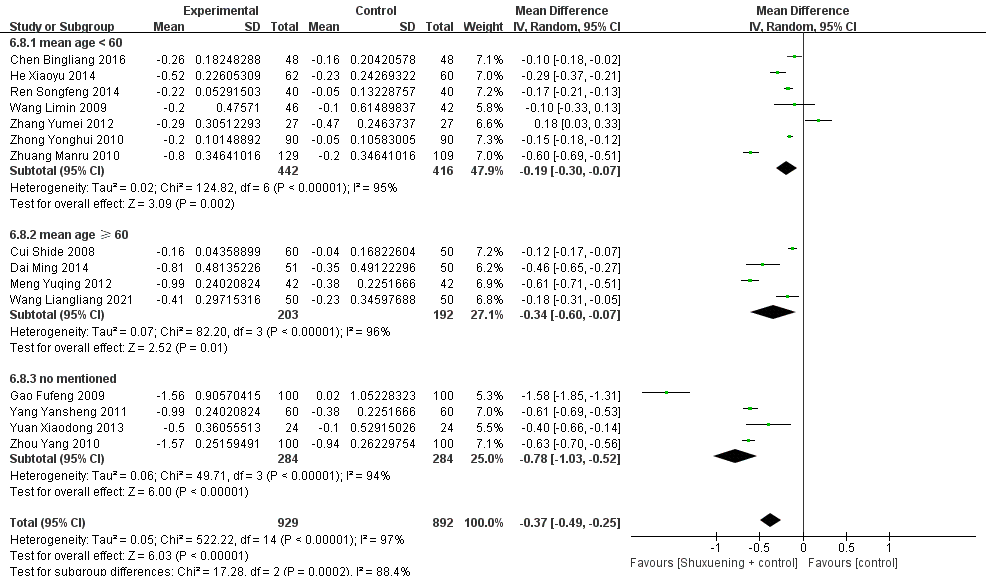


**Fig. S14 Effect of SXN on HCT.**

(A) Forest plot of HCT. (B) Sensitivity analysis revealed the reliability of result.


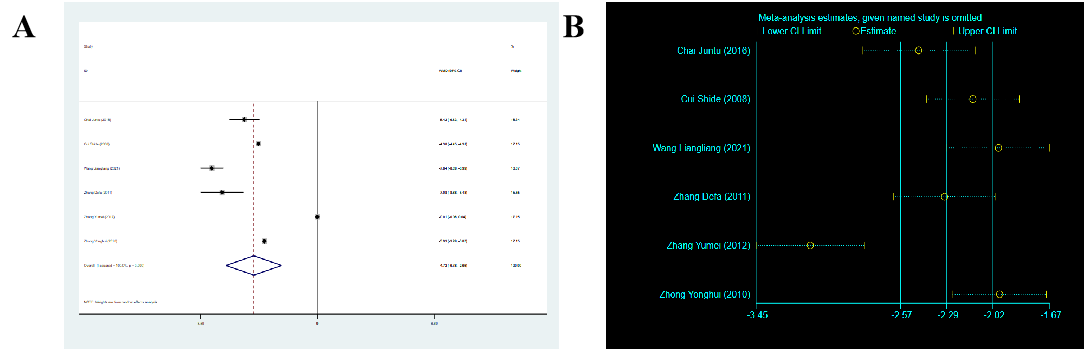


**Fig. S15 Effect of SXN on HS.**

(A) Forest plot of HS. (B) Sensitivity analysis revealed the reliability of result.


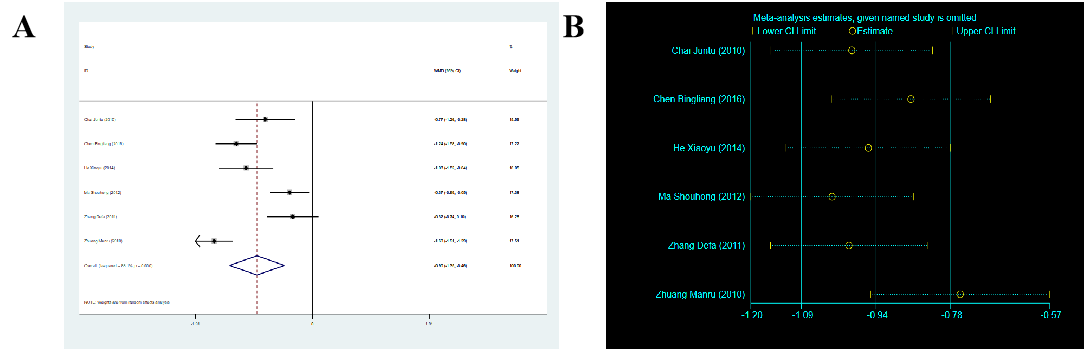

Supplement: Supplementary file 1 [file DataSheet1.docx]
